# Concealing Untrustworthiness: The Role of Conflict Monitoring in a Social Deception Task

**DOI:** 10.3389/fpsyg.2021.718334

**Published:** 2021-08-20

**Authors:** Fee-Elisabeth Hein, Anja Leue

**Affiliations:** Institute of Psychology, Faculty of Arts, University of Kiel, Kiel, Germany

**Keywords:** deception, conflict monitoring, N2, CIT, trustworthiness

## Abstract

Deception studies emphasize the important role of event-related potentials (ERPs) to uncover deceptive behavior based on underlying neuro-cognitive processes. The role of conflict monitoring as indicated by the frontal N2 component during truthful and deceptive responses was investigated in an adapted Concealed Information Test (CIT). Previously memorized pictures of faces should either be indicated as truthfully trustworthy, truthfully untrustworthy or trustworthy while concealing the actual untrustworthiness (untrustworthy-probe). Mean, baseline-to-peak and peak-to-peak amplitudes were calculated to examine the robustness of ERP findings across varying quantification techniques. Data of 30 participants (15 female; age: *M* = 23.73 years, *SD* = 4.09) revealed longer response times and lower correct rates for deceptive compared to truthful trustworthy responses. The frontal N2 amplitude was more negative for untrustworthy-probe and truthful untrustworthy compared to truthful trustworthy stimuli when measured as mean or baseline-to-peak amplitude. Results suggest that deception evokes conflict monitoring and ERP quantifications are differentially sensitive to a-priori hypotheses.

## Introduction

Research addressing the underlying neuro-cognitive processes of deception emphasizes the promising role of cognitive-motivational processes and event-related potentials (ERP) of the electroencephalogram (EEG) to differentiate between truthful and deceptive behavior (Johnson et al., 2004, 2008; Wu et al., 2009; Leue et al., 2012a; Meijer et al., 2014; Leue and Beauducel, 2015, 2019; Fu et al., 2017). If neuro-cognitive processes of deception could be successfully disentangled in EEG research, its application for social situations might be probed. Most common contents of deception in everyday life concern personal feelings, attitudes, and opinions (DePaulo et al., 2003). Sometimes deception can serve to facilitate social interactions, for example, by withholding personal opinions that would promote disagreements or that would hurt feelings. When deception goes along with more serious consequences, it would be in interest of a society, to uncover deceptive behavior if legal regulations in judicial trials allow for a differentiation of deception and truth by means of EEG data and if validation of this differentiation could be sufficiently confirmed.

Deception is argued to be a cognitively demanding process and a conflict-inducing behavior. As honest communication is a basic prerequisite for a well-functioning social interaction and is deeply anchored in social values, a stimulus that requires a deceptive response can induce a cognitive conflict between the default tendency to tell the truth and the requirement to deceive. It involves the retention of existing knowledge, the suppression of the tendency to respond truthfully and the invention or activation of an alternative response (Debey et al., 2014; Walczyk et al., 2014). These processes require not only working memory capacity and mental effort by involving executive functions, but also conflict monitoring and cognitive control (Langleben et al., 2002; Johnson et al., 2004; Nunez et al., 2005; Wu et al., 2009; Leue et al., 2012a, 2014; Fu et al., 2017; Koeckritz et al., 2019). According to the (integrative) conflict monitoring hypothesis (Botvinick et al., 2001; Botvinick, 2007) conflict monitoring takes place in the anterior cingulate cortex (ACC). The ACC sends out information when a conflict is detected to increase cognitive control to minimize the occurrence of further conflicts. Thereby the occurrence of a conflict serves as a teaching signal leading the selection of task solving strategies (Botvinick, 2007). The simultaneous activation of competing response options, which requires the overriding of dominant action tendencies that are not goal-directed for the task, elicits a conflict. The conflict arises between the processing paths that lead to correct or incorrect responses as defined by task instructions (Botvinick et al., 2001; Yeung et al., 2004; Botvinick, 2007). It has been assumed that the level of conflict increases as the absolute activation of the representations of the competing response options increase and as the number of competing representations increases. Moreover, the level of conflict reaches a maximum when the activation level of competing representations is equal (Berlyne, 1957; Botvinick et al., 2001).

One component of the ERP that has been of interest in the context of uncovering deception is the N2. The N2 is a stimulus-locked negative-going component that occurs on fronto-central recording sites (Fz, FCz, Cz) and peaks around 200–350 ms after stimulus presentation (Nieuwenhuis et al., 2003; Amodio et al., 2008; Folstein and van Petten, 2008). There is evidence that the N2 is generated in the ACC and is therefore related to cognitive control and conflict monitoring in cognitively demanding tasks (Amodio et al., 2008; Leue et al., 2012c, 2014) like deception. Consistently, deception studies suggest that the amplitude of the N2 is more negative after evaluating a stimulus requiring a deceptive response compared to a truthful response (Wu et al., 2009; Fu et al., 2017). The current study investigates the role of conflict monitoring as indicated by the frontal N2 amplitude in a deception paradigm. In particular, we will examine whether the amount of conflict monitoring increases the more convinced subjects are about a social attribute (e.g., trustworthiness) they should conceal. Behavioral evidence further supports the assumption that deception is a cognitively demanding process. Deceptive responses compared to truthful responses are usually associated with longer response times and a higher error rate (Meijer et al., 2007; Hu et al., 2011; Leue et al., 2012a; Suchotzki et al., 2015, 2017; Fu et al., 2017).

Previously, different ERP quantification methods such as baseline-to-peak amplitude (e.g., Hu et al., 2011) or combined amplitude and latency measurement (e.g., Gamer and Berti, 2010) were selected to investigate the N2 during deception. Therefore, we aimed at investigating the robustness of our N2 findings for different ERP quantifications such as mean, baseline-to-peak, and peak-to-peak amplitudes (Luck, 2014). Leue et al. (2013) found that the frontal N2 in a go/nogo task can be measured more reliably as a mean amplitude vs. baseline-to-peak amplitude depending on filtering and number of nogo epochs. In contrast, Kleene et al. (2021) revealed a higher reliability of the frontal N2 in an adapted CIT when measured as baseline-to-peak or peak-to-peak amplitude than measured as mean amplitude. Thus, depending on the experimental paradigm quantification of ERPs matters for ERP reliability. Moreover, less is known whether empirical findings in a deception paradigm generalize across ERP quantifications. Therefore, the generalizability and robustness of ERP findings across varying quantification techniques contribute to the validation of experimental findings in a deception paradigm. Three different quantification methods (mean, baseline-to-peak, and peak-to-peak amplitude) will be used to report the experimental findings for the N2 amplitude in an adapted social deception task.

### CITs and Adaptions

A frequently used paradigm to study processes underlying deception by means of concealing knowledge is the Concealed Information Test (originally Guilty Knowledge Test, GKT; Lykken, 1959, 1960). The original CIT differentiates two stimulus types, namely probes and irrelevants (later on three types: probe, target, irrelevants; see Rosenfeld et al., 1988). Probes are relevant items that are familiar to the subjects who are supposed to conceal the familiarity. Irrelevants are unfamiliar to the subjects who are supposed to truthfully indicate the unfamiliarity. This original CIT follows the assumption that probes have a special significance (i.e., stimulus salience) for subjects concealing their knowledge of these items (for argumentations on orienting response see Klein Selle et al., 2016, 2017). Thereby, familiar stimuli compared to unfamiliar stimuli are more salient, as manifested in a more pronounced P3 component following probe compared to irrelevant stimuli (Verschuere et al., 2011; Leue and Beauducel, 2015, 2019). To investigate other cognitive processes underlying deception (e.g., mental effort) an adaption of the CIT with a specific experimental condition is an appropriate tool (Koeckritz et al., 2019; Leue and Beauducel, 2019). Koeckritz et al. (2019) investigated stimulus salience and mental effort (i.e., the cognitive capacity that remains when stimuli are processed) during deception within one paradigm by means of the P3. Subjects were instructed to conceal knowledge of faces (probe) in relation to unknown faces (irrelevant) in one task condition. In another task condition, all stimuli (faces with a social attribute, namely trustworthiness) were known prior to the task. For the trustworthiness condition, the stimuli were classified into three categories. (1) Following *probes* (i.e., untrustworthy stimuli), subjects are supposed to give a deceptive answer (i.e., to indicate the face would be trustworthy). (2) Following “*trustworthy”* stimuli, subjects are supposed to indicate the attribute truthfully. (3) “*Untrustworthy”* stimuli have an opposing attribute expression to trustworthy stimuli and subjects are asked to indicate the attribute truthfully. Koeckritz et al. (2019) assigned the faces to the pre-defined attribute categories randomly (see section 1.3 in Koeckritz et al., 2019). Deception can induce successive cognitive processes like conflict monitoring occurring prior to the investment of P3-related mental effort. Conflict monitoring as reflected by the frontal N2 amplitude (Gamer and Berti, 2010; Fu et al., 2017) was found to be more negative after stimuli requiring a deceptive vs. truthful response in previous studies (Wu et al., 2009; Fu et al., 2017). Therefore, we assume the frontal N2 to be more negative after probes compared to truthful trustworthy or truthful untrustworthy faces with instruction-conform responses (hypothesis 1).

### Trustworthiness

Social interactions with an unfamiliar person start with a first impression of the person that is received within a very short period of time (Willis and Todorov, 2006; Todorov et al., 2009; Yang et al., 2011). The first impression includes, among other social and/or physical attributes, the evaluation of a person's trustworthiness (Willis and Todorov, 2006; Engell et al., 2007; Marzi et al., 2014; Meconi et al., 2014). Trustworthiness as a social attribute has a significant impact on the likelihood and kind of engaging in social interactions with the evaluated person (Yang et al., 2011). Moreover, trustworthiness might be concealed in social interactions when especially untrustworthiness could not be communicated without breaking social rules or expectations. Therefore, the false or concealed evaluation of a person as trustworthy can be associated with unpleasant consequences and a stronger cognitive and moral conflict than its truthful declaration. Following Koeckritz et al. (2019), untrustworthiness of faces is the characteristic to be deceptively responded to in the current study. Assuming that the extent of required conflict monitoring is greater when deceiving about self-related facts vs. non-self-related facts (Nunez et al., 2005; Hu et al., 2011), it can be hypothesized that the amount of personal involvement (e.g., through intensity of individual beliefs, norms, and values) also has an impact on the amount of conflict monitoring required for successful deception. In terms of untrustworthiness, this would mean that the less trustworthy an individual judges a face, the stronger the conflict monitoring should be between the representations of a truthful (untrustworthy) and a deceptive (trustworthy) response. Thus, deception should elicit more conflict monitoring in subjects the greater the discrepancy between actual perceived expressions of trustworthiness and the deceptive response. This assumption is consistent with the argumentation of Berlyne (1957) and Botvinick et al. (2001), who suggest in the context of the conflict-monitoring hypothesis that the conflict level increases with the absolute activation level of competing representations and becomes maximal when they are equal. To investigate the influence of the absolute activation level, the affective vs. neutral variation of the presented faces was introduced as another experimental variation on the conflict monitoring intensity. Affective intensity of the faces has been evaluated by means of pre-ratings (Ma et al., 2015). That is, in addition to the stimulus-related conflict between probes and truthful (un-)trustworthy faces, we introduce the affective compared to the neutral condition of the faces as another operationalization of conflicting or competing representations as presumed by Botvinick et al. (2001) for non-deception tasks. Overall, we hypothesize larger (more negative) frontal N2 amplitudes following untrustworthy-probes compared to truthful stimuli in the affective vs. neutral condition (hypothesis 2).

### Aims and Hypotheses

This study aims at investigating cognitive processes underlying deception to the social attribute (un)trustworthiness by means of the frontal N2 amplitude. An adapted CIT has been used to examine conflict monitoring during deception. It has been assumed that the evaluation of untrustworthy-probe stimuli elicits a more negative N2 compared to truthful trustworthy or truthful untrustworthy stimuli (hypothesis 1: Picture type main effect). To investigate the intensity of the participants' perception of (un)trustworthiness on the conflict monitoring intensity, we selected stimulus material that had been judged as neither trustworthy nor untrustworthy (neutral condition) or as particularly trustworthy or untrustworthy (affective condition). The discrepancy between perceived and reported untrustworthiness in untrustworthy-probes is larger in the affective vs. neutral condition, which is assumed to enhance conflict monitoring. We hypothesized a more negative stimulus-related N2 following untrustworthy-probes compared to truthful (un-)trustworthy pictures in the affective vs. neutral condition (hypothesis 2: Picture type × Condition interaction). Furthermore, we examined whether the results for the N2 vary across task sequence (affective-neutral vs. neutral-affective condition; Picture type × Task sequence interaction) and Gender of the participant (Picture type × Gender interaction). To compare the results for the frontal N2 in an adapted CIT, three quantification methods (mean-, baseline-to-peak-, peak-to-peak amplitude) were performed to investigate the robustness of the experimental findings for hypothesis 1 and 2. A more comprehensive understanding of the role of conflict monitoring during deception helps to extend the theoretical understanding of deception.

## Methods

### Participants

Participants were recruited through websites, Facebook groups and mailing lists of student groups of the University of Kiel (Germany). A total of 73 right-handed students of the University of Kiel, Germany (38 female; age: *M* = 23.88 years, *SD* = 4.17) took part in the study. Thirty-six participants had to be excluded because no response port codes were sent in the initial phase of the study, which would have been important to disentangle epochs with correct and incorrect responses. These participants were used as a pilot sample for paradigm optimization. Of the 37 participants remaining for the present study, seven other participants had to be excluded due to an insufficient number of artifact-free epochs (<25 epochs; see Kleene et al., 2021) per picture type after conducting the Independent Component Analysis (ICA; see below). A *post-hoc* analysis with G^*^Power, version 3.1.9.4 (Faul et al., 2007) with an intended power of 0.80 and a significance level of 0.05, two-tailed, and for a presumed effect size *f* of 0.25 revealed an optimal sample size of 28 participants for an ANOVA F-test with repeated measures within-between interaction. According to Cohen (1988), effect size *d* can be transferred into *f* because *f* is applied as an effect size in G^*^Power 3.1.9.4. The final sample consisted of 30 students (15 female; age: *M* = 23.73 years, *SD* = 4.09). All participants reported normal or corrected to normal vision. Due to the significant reduction of the sample, the study design was reduced to an experimental design without differential psychological variables. The study was confirmed by the Ethics Committee of the Medical Faculty of Kiel University, Germany (May, 2019).

### Adapted CIT

Twenty-four pictures of faces with neutral facial expressions were taken from the Chicago Face Database (CFD), which provides norming data and subjective ratings such as trustworthiness of independent judges for each picture (Ma et al., 2015). In order to balance cultural and gender effects in the stimulus material, 12 female and 12 male faces of the white ethnic group were chosen.

The adapted CIT contained two blocks, implementing an affective and a neutral condition, and three types of pictures. *Truthful trustworthy* pictures show trustworthy faces whose trustworthiness should be truthfully indicated by button press (right arrow). *Truthful untrustworthy* pictures show untrustworthy faces whose trustworthiness should be truthfully indicated by button press (left arrow). *Untrustworthy-probe* pictures show untrustworthy faces whose untrustworthiness should be concealed by participants, who instead indicate trustworthiness (right arrow). The assignment of trustworthiness to the response keys was not counterbalanced across participants. In the affective condition, faces were assigned to the picture types according to their trustworthiness score in the pre-rating of Ma et al. (2015). Thereby, faces of the two most trustworthy men and women according to the pre-rating of the CFD (Ma et al., 2015) were selected as truthful trustworthy pictures, while faces of the four least trustworthy men and women were randomly assigned to the truthful untrustworthy or untrustworthy-probe categories (Table 1). This assignment of the pictures to the picture types was constant across participants.

**Table 1:** Assignment of stimuli to picture types and their rated trustworthiness.

	**Pre-rating CFD^a^: male pictures**	**Pre-rating CFD^a^: female pictures**	**Pre-rating CFD^a^: all pictures**	**Current rating: male pictures**	**Current rating: female pictures**	**Current rating: all pictures**
*Affective condition*						
Untrustworthy-probe [CFD picture numbers: WM-010, WM-243, WF-239, WF-210]	2.28 (1.38)	2.74 (1.55)	2.51 (1.47)	1.93 (0.90)	2.50 (1.20)	2.22 (1.05)
Truthful trustworthy [CFD picture numbers: WM-257, WM-252, WF-242, WF-233]	3.91 (1.05)	4.19 (1.38)	4.05 (1.21)	5.39 (1.96)	5.14 (1.10)	5.27 (1.04)
Truthful untrustworthy [CFD picture numbers: WM-249, WM-220, WF-222, WF-237]	2.40 (1.44)	2.84 (1.44)	2.62 (1.44)	2.32 (1.38)	3.60 (1.46)	2.96 (1.42)
*Neutral condition*						
Untrustworthy-probe [CFD picture numbers: WM-251, WM-006, WF-221, WF-202]	3.42 (1.38)	3.42 (1.40)	3.42 (1.39)	4.56 (1.39)	3.57 (1.28)	4.06 (1.33)
Truthful trustworthy [CFD picture numbers: WM-040, WM-018, WF-241, WF-034]	3.41 (1.24)	3.40 (1.33)	3.40 (1.28)	4.01 (1.33)	4.39 (1.40)	4.20 (1.36)
Truthful untrustworthy [CFD picture numbers: WM-230, WM-031, WF-009, WF-245]	3.39 (1.45)	3.42 (1.29)	3.40 (1.37)	3.97 (1.38)	3.87 (1.55)	3.92 (1.46)

a *Ma et al. (2015). Ratings of trustworthiness were assessed using a 7-point likert scale from 1 = not at all trustworthy to 7 = very strongly trustworthy. Rating scores are presented in mean (standard deviation, SD). Each picture type was presented 50 times per experimental block. Since each picture type consists of 4 pictures, the presentation frequencies between the individual pictures vary from 12 to 13. In the affective experimental block, pictures W-239, WF-210, WM-252, WF-242, WM-249, WF-237 were presented 13 times, pictures WM-010, WM-243, WM-257, WF-233, WM-220, WF-222 were presented 12 times. In the neutral experimental block, pictures WM-251, WM-006, WM-040, WM-018, WM-230, WM-031 were presented 13 times, pictures WF-221, WF-202, WF-241, WF-034, WF-009, WF-245 were presented 12 times*.

In the neutral condition, the six male and six female faces with the most neutral expression of trustworthiness according to the pre-rating of the CFD were selected and randomly assigned to truthful trustworthy, truthful untrustworthy and untrustworthy-probe categories (Table 1). Results of a manipulation check using independent two-sample *t*-tests revealed that in total the current sample rated the faces marginally more trustworthy than the CFD sample, *t*_(78)_ = 1.82, *p* = 0.07 (https://www.graphpad.com/quickcalcs/ttest1/?Format=SD). The number of raters per picture varied between *n* = 23 and 94 in the CFD (Ma et al., 2015) and was constant with *n* = 37 raters in the current study. Looking at the particular picture types, in the affective condition the current sample rated the truthful trustworthy picture type significantly more trustworthy than the CFD sample, *t*_(61)_ = 4.28, *p* < 0.01. In the neutral condition, the current sample rated all picture types at least marginally more trustworthy than the CFD sample [truthful trustworthy: *t*_(94)_ = 2.91, *p* = 0.01; truthful untrustworthy: *t*_(94)_ = 1.76, *p* = 0.08; untrustworthy-probes: *t*_(77)_ = 2.08, *p* = 0.04]. Trustworthiness ratings obtained from the current sample differed significantly between the affective and neutral conditions for all picture types [truthful trustworthy: *t*_(36)_ = 6.37, *p* < 0.01; truthful untrustworthy: *t*_(36)_ = 6.49, *p* < 0.01; untrustworthy-probes: *t*_(36)_ = 14.75, *p* < 0.01]. As expected, the faces of the present study were mostly rated significantly more trustworthy or untrustworthy in the affective compared to the neutral condition. For descriptives see Table 1.

Instructions were presented in white letters (Arial, 19 pt) on a black 17″ flat screen. In a 7-min learning phase, all 12 faces of the respective condition were presented with three headings indicating the picture type. A practice series involved 15 trials (5 of each picture type). If more than three errors were committed during the 15 practice trials, the instructions were shown again and the practice trials were repeated. In the experimental block participants performed 150 trials (50 of each picture type; 1:1:1 ratio between untrustworthy-probes, truthful trustworthy and truthful untrustworthy) presented in a pseudorandomized order. After a 2-min break separating the two experimental blocks, the procedure was repeated for the second condition. To control for sequence effects, the task sequence affective-neutral (*n* = 15) vs. neutral-affective (*n* = 15) was pseudo-randomly balanced across participants.

**Figure 1:**
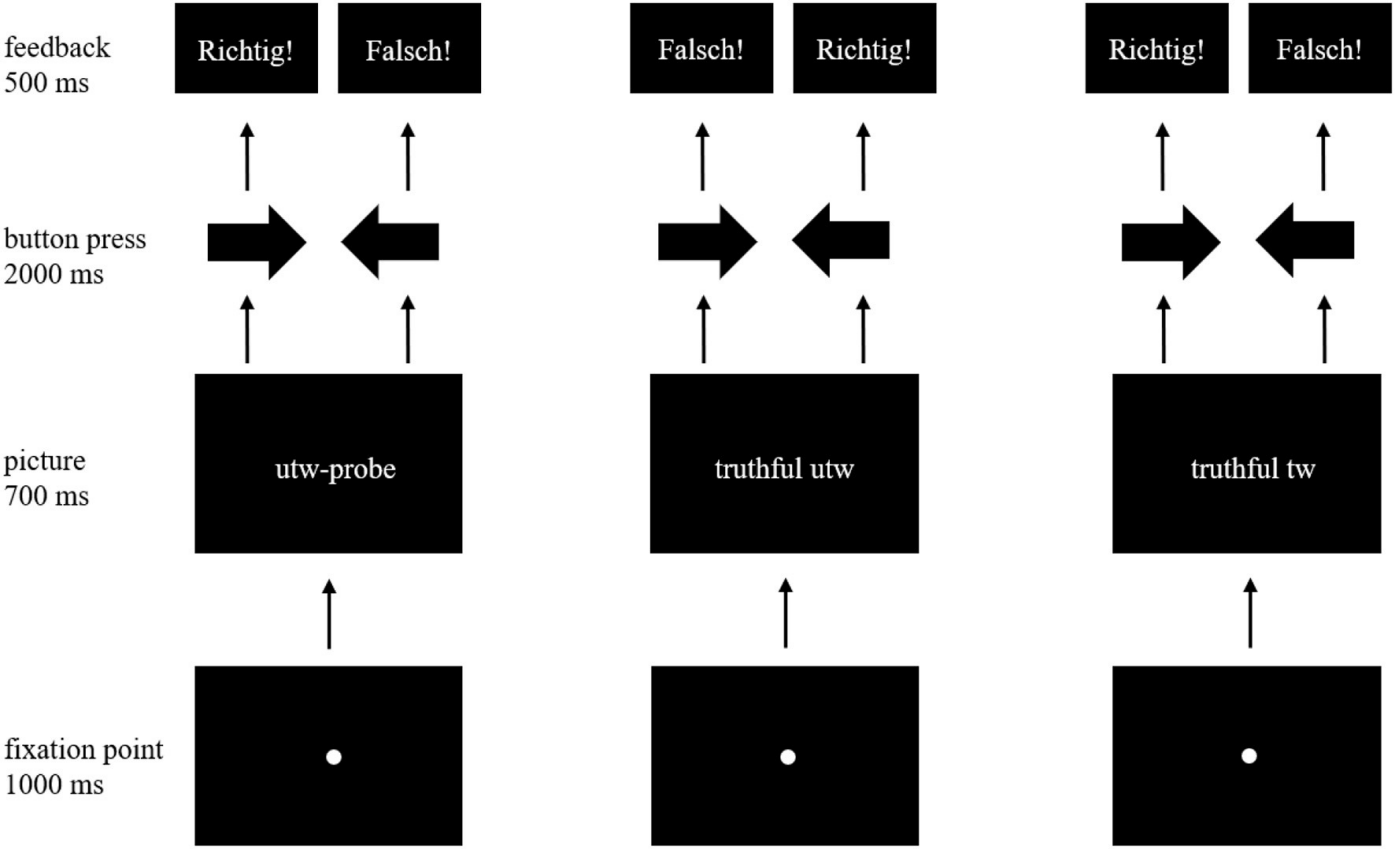
Trial sequence for one item of each picture type (untrustworthy-probe, truthful untrustworthy, truthful trustworthy). The affective and neutral condition follow the same trial sequence and differ only in the stimulus material. For stimulus material please see Table 1 and the Chicago Face Database (Ma et al., 2015). Inter-trial-intervals are not presented in this figure. The feedback “Richtig!” means “correct!” in German, “Falsch!” means “wrong!”.

Each trial sequence (Figure 1) started with a fixation point which was presented in the center of the screen for 1,000 ms followed by a picture (picture size: 611 × 429.5 pixels) presented for 700 ms. After the picture disappeared, the screen remained black and the participant had a maximum of 2,000 ms to indicate the trustworthiness of the face by pressing the left (untrustworthy) or right (trustworthy) arrow key in accordance with instruction. Feedback was provided according to the actual performance immediately after the response for 500 ms, whereby the word “Richtig!” (German for “correct”) appeared on the screen, if the participant had responded according to the instructions in the predefined time interval. Otherwise, the word “Falsch!” (German for “wrong”) appeared. The inter-trial-interval (ITI) varied in a pseudorandomized order between 1,000, 1,500, and 2,000 ms. During an ITI the screen remained black. The trial sequence was identical to the study of Koeckritz et al. (2019), but with differences in stimulus material, number of pictures and trials, and feedback.

### Procedure

After arriving, participants gave written informed consent. They were placed at a distance of ~70 cm in front of the screen and were prepared for EEG recording. For the presentation of the adapted CIT Presentation V18.1 (Neurobehavioral Systems, Albany, NY, USA) was used. The completion of the task, including learning, practice, and experimental phases, took on average 50 min. After completion of the adapted CIT the manipulation check was conducted, and participants completed the figural (numbers of task group: 7–9) and memory test (numbers of task group: 10–11) of the German Intelligenz-Struktur-Test 2000 R (Liepmann et al., 2007) and the German BIS/BAS scales (Strobel et al., 2001). The results of the personality traits are not reported here as the sample size was too small for testing hypotheses on trait variables. The whole examination lasted 2.5 h on average. At the end of the examination, remaining questions were answered, the participants were thanked and given a course credit of 2.5 h or 20€.

### EEG Recording

**Figure 2:**
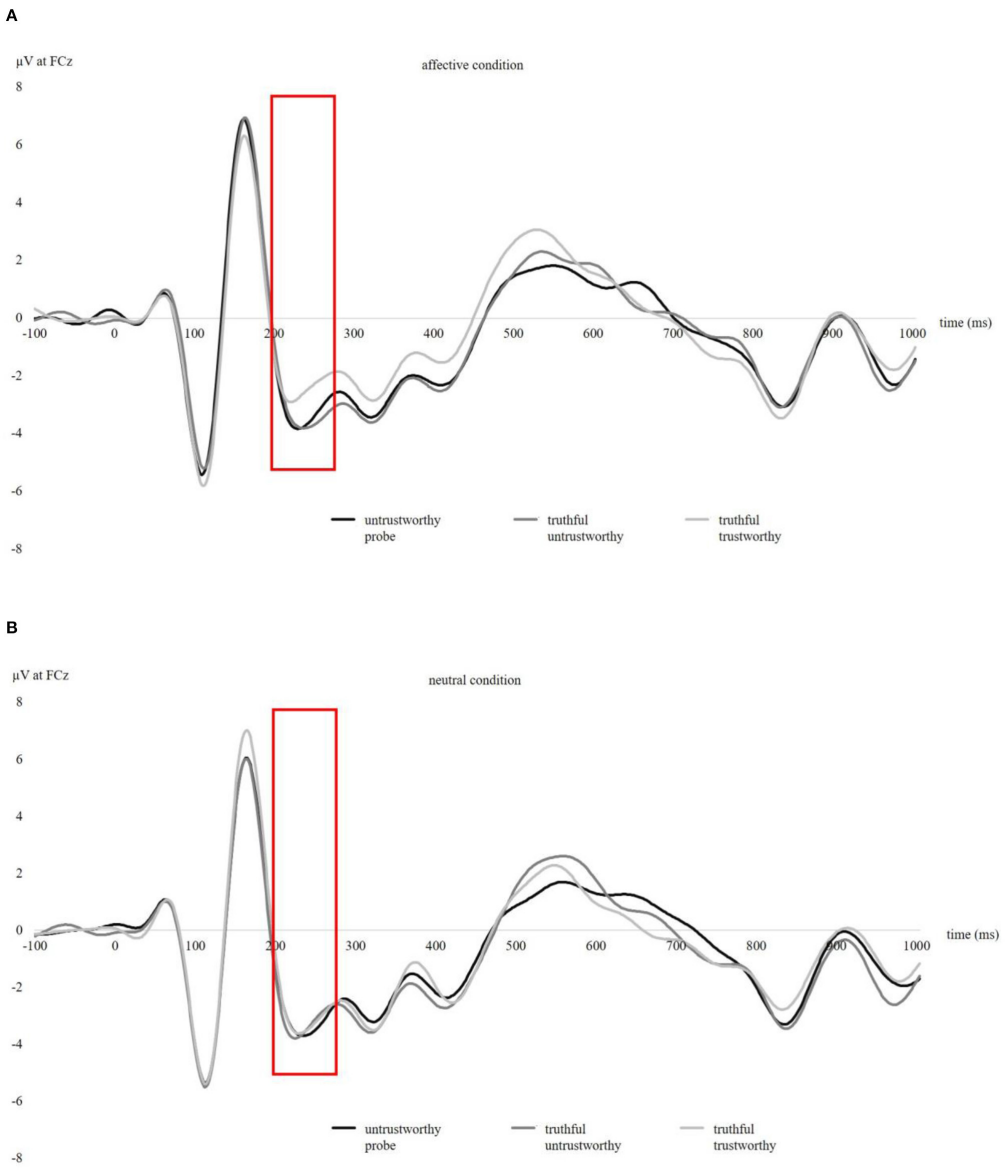
Stimulus-locked grand averages at FCz from 0 to 1,000 ms post-stimulus, with a 100 ms pre-stimulus baseline for three picture types (untrustworthy-probe, truthful untrustworthy, truthful trustworthy) in the affective condition **(A)** and neutral condition **(B)**. Stimulus was presented from 0 to 700 ms. N2 amplitudes were identified between 200 and 275 ms post-stimulus.

The EEG recording and pre-processing were performed in accordance with recommended guidelines for EEG research (Picton et al., 2000; Keil et al., 2014). EEG was recorded with 64 active scalp electrodes arranged according to the 10/20 system (Jasper, 1958) using the ActiveTwo EEG system (BioSemi, Amsterdam, Netherlands). The electrooculogram (EOG) was recorded from two additional horizontal electrodes placed beyond the epicanthi of both eyes and one vertical electrode located ~1 cm below the right eye. The ground electrode was formed by the Common Mode Sense active electrode (CMS) and the Driven Right Leg passive electrode (DRL). The signals were digitized at 512 Hz sampling rate on a computer using ActiView software (BioSemi). The impedances were kept within ±30 kΩ during EEG recording. Offline analysis was conducted using EEGLab v2019.1 (Delorme and Makeig, 2004) based on MATLAB 9.8.0 (The MathWorks). Prior to ERP analysis, an offline high-pass filter of 1 Hz and an offline low-pass filter of 15 Hz were applied (Amodio et al., 2008; Leue et al., 2013). The average signal of the mastoids (P9 and P10) was used to re-reference the data. Independent component analysis (ICA) was applied to correct for ocular artifacts (Mognon et al., 2011). Epochs containing further technical and muscular artifacts were rejected when the EEG signal exceeded ±85 μV. Grand averages (0–1,000 ms post-stimulus, with a 100 ms pre-stimulus baseline) indicate a N2 amplitude between 200 and 275 ms post-stimulus at FCz (Figure 2; for grand averages at Fz, Cz, and Pz see Supplementary Figures 1–3) and a P2 amplitude between 135 and 200 ms post-stimulus for the calculation of the N2 peak-to-peak amplitude. Following Kleene et al. (2021), the N2 was quantified as mean amplitude, baseline-to-peak amplitude (the most negative peak in the defined time interval), and peak-to-peak amplitude (measured as baseline-to-peak N2 minus baseline-to-peak P2) to allow a comparison of quantification methods (Luck, 2014).

### Statistical Analysis

IBM SPSS (Version 25) was used for statistical analyses. Separate repeated measures ANOVAs were performed for behavioral data including percentage of correct responses and response times, for ERP data including the mean N2 amplitudes, the baseline-to-peak N2 amplitudes and the peak-to-peak N2 amplitudes. Picture type and Condition (affective vs. neural) were applied as repeated-measures factors in ANOVAs for behavioral data and N2 amplitudes. Furthermore, Electrode position (Fz, FCz, Cz, Pz) was included as a repeated-measures factor in the ANOVAs for N2 amplitudes. To examine whether the N2 and behavioral data vary depending on gender and task sequence, Task sequence (affective-neutral vs. neutral-affective condition) and Gender of the participants were applied as between-subject factors in ANOVAs for behavioral data and N2 amplitudes to perform a statistical design that corresponds to Koeckritz et al. (2019). Whenever the sphericity assumption was violated (e.g., numerator df ≥ 2), Greenhouse-Geisser correction was used and Greenhouse-Geisser epsilon (ε) is reported along with uncorrected degrees of freedom. In addition to *p*-values that are presented for (two-tailed) tests, partial eta square (η_*p*_^2^) is reported to evaluate the effect size. For the comparison of the quantification methods (mean, baseline-to-peak and peak-to-peak amplitude) effect sizes were transformed from η_*p*_^2^ to effect size *d* into coefficient *r*. Transformation was conducted with an online calculator from Psychometrica (https://www.psychometrica.de/effect_size.html#transform).

## Results

### Mean N2 Amplitude

A significant main effect of Position was observed, *F*_(3, 78)_ = 10.22, *p* < 0.01, η_*p*_^2^ = 0.28, ε = 0.37. Simple contrasts revealed a more negative N2 amplitude at FCz compared to Fz, *F*_(1, 26)_ = 7.25, *p* = 0.01, η_*p*_^2^ = 0.22, and compared to Pz, *F*_(1, 26)_ = 13.63, *p* < 0.01, η_*p*_^2^ = 0.34, and a more negative N2 amplitude at Fz compared to Pz, *F*_(1, 26)_ = 5.68, *p* = 0.03, η_*p*_^2^ = 0.18 (Table 2, for an overview of all N2 amplitudes for the main and interaction effects of Picture type, Condition, and Task sequence, see Supplementary Tables 1–4). Furthermore, there was a significant Picture type main effect, *F*_(2, 52)_ = 5.23, *p* = 0.01, η_*p*_^2^ = 0.17, ε = 0.94. Simple contrasts revealed a significantly more negative N2 amplitude for untrustworthy-probes compared to truthful trustworthy pictures, *F*_(1, 26)_ = 7.01, *p* = 0.01, η_*p*_^2^ = 0.21, and for truthful untrustworthy pictures compared to truthful trustworthy pictures, *F*_(1, 26)_ = 8.27, *p* = 0.01, η_*p*_^2^ = 0.24, but not for untrustworthy-probe compared to truthful untrustworthy pictures, *F*_(1, 26)_ = 0.64, *p* = 0.43 (Table 2). There were no significant interactions of Picture type × Condition, *F*_(2, 52)_ = 1.37, *p* = 0.26, ε = 0.86, Picture type × Task sequence, *F*_(2, 52)_ = 1.45, *p* = 0.24, ε = 0.94, or Picture type × Gender, *F*_(2, 52)_ = 0.47, *p* =. 62, ε = 0.94 (for the results of a *post-hoc* exploration of all remaining main effects, two-way interactions and higher-order interactions see Supplementary Table 4).

**Table 2:** N2 amplitudes in microvolt (μV) for electrode position and picture type based on different quantifications.

	**Mean amplitude**	**Baseline-to-peak amplitude**	**Peak-to-peak amplitude N2-P2**
Electrode position^a^			
Fz	−2.43 (3.56) [0.65]	−4.71 (3.86) [0.70]	−12.57 (6.58) [1.20]
FCz	−3.00 (3.43) [0.63]	−5.27 (3.69) [0.67]	−13.08 (6.94) [1.27]
Cz	−2.95 (3.35) [0.61]	−5.31 (3.55) [0.65]	−12.40 (7.18) [1.32]
Pz	−0.26 (3.83) [0.70]	−2.58 (3.59) [0.66]	−7.50 (5.74) [1.05]
Picture type^b^			
Untrustworthy-probe	−3.13 (3.38) [0.62]	−5.45 (3.65) [0.67]	−13.19 (7.07) [1.29]
Truthful untrustworthy	−3.25 (3.72) [0.68]	−5.42 (4.07) [0.74]	−13.18 (7.14) [1.30]
Truthful trustworthy	−2.64 (3.44) [0.63]	−4.95 (3.60) [0.66]	−12.87 (6.76) [1.23]

*Scores are presented in mean (standard deviation, SD) [standard error of the mean, SEM]*.

a *Amplitudes are averaged over picture types and conditions*.

b *Amplitudes at FCz averaged over the conditions (affective vs. neutral)*.

### Baseline-To-Peak N2 Amplitude

**Figure 3:**
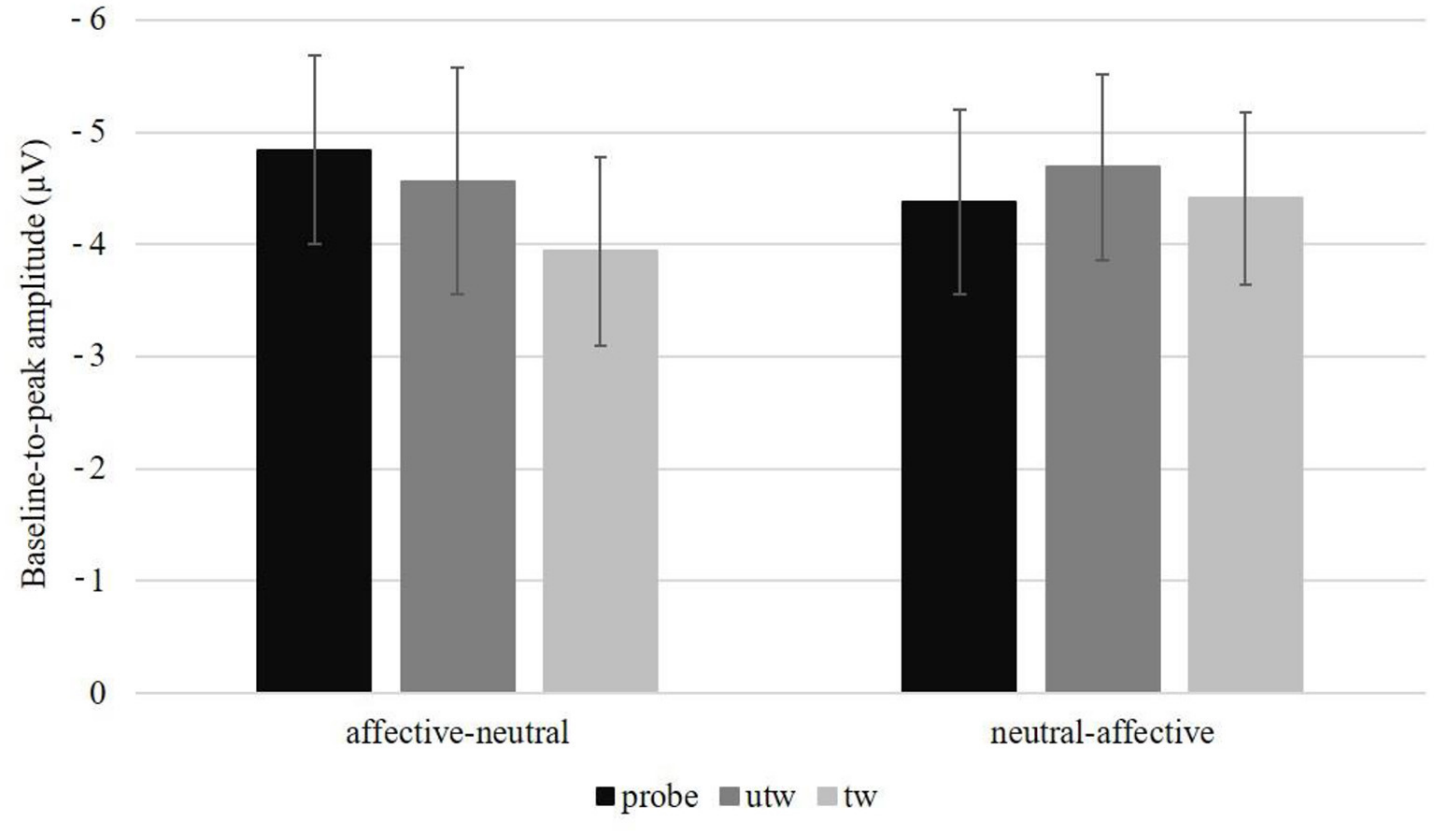
Baseline-to-peak amplitude in microvolt (μV) and standard errors averaged over electrode positions (Fz, FCz, Cz, Pz) per picture type and task sequence (affective-neutral vs. neutral-affective). probe, untrustworthy-probe; utw, truthful untrustworthy; tw, truthful trustworthy.

A significant main effect of Position was observed, *F*_(3, 78)_ = 10.90, *p* < 0.01, η_*p*_^2^ = 0.30, ε = 0.39. Simple contrasts revealed a more negative N2 amplitude at FCz compared to Fz, *F*_(1, 26)_ = 6.22, *p* = 0.02, η_*p*_^2^ = 0.19, and compared to Pz, *F*_(1, 26)_ = 14.37, *p* < 0.01, η_*p*_^2^ = 0.36 and a more negative N2 amplitude at Fz compared to Pz, *F*_(1, 26)_ = 6.09, *p* = 0.02, η_*p*_^2^ = 0.19 (Table 2). Furthermore, there was a significant Picture type main effect, *F*_(2, 52)_ = 3.30, *p* < 0.05, η_*p*_^2^ = 0.11, ε = 0.93. Simple contrasts revealed a significantly more negative N2 amplitude for untrustworthy-probes compared to truthful trustworthy pictures, *F*_(1, 26)_ = 6.11, *p* = 0.02, η_*p*_^2^ = 0.19, and for truthful untrustworthy pictures compared to truthful trustworthy pictures, *F*_(1, 26)_ = 5.18, *p* = 0.03, η_*p*_^2^ = 0.17, but not for untrustworthy-probe compared to truthful untrustworthy pictures, *F*_(1, 26)_ = 0.01, *p* = 0.92 (Table 2). We found a marginal interaction of Picture type × Task sequence, *F*_(2, 52)_ = 2.75, *p* = 0.08, η_*p*_^2^ = 0.10, ε = 0.93 (Figure 3). There were no significant interactions of Picture type × Condition, *F*_(2, 52)_ = 0.01, *p* = 0.99, ε = 0.99, or Picture type × Gender, *F*_(2, 52)_ = 0.24, *p* = 0.77, ε = 0.93 (for the results of a *post-hoc* exploration of all remaining main effects, two-way interactions and higher-order interactions see Supplementary Table 4).

### Peak-To-Peak N2 Amplitude

**Figure 4:**
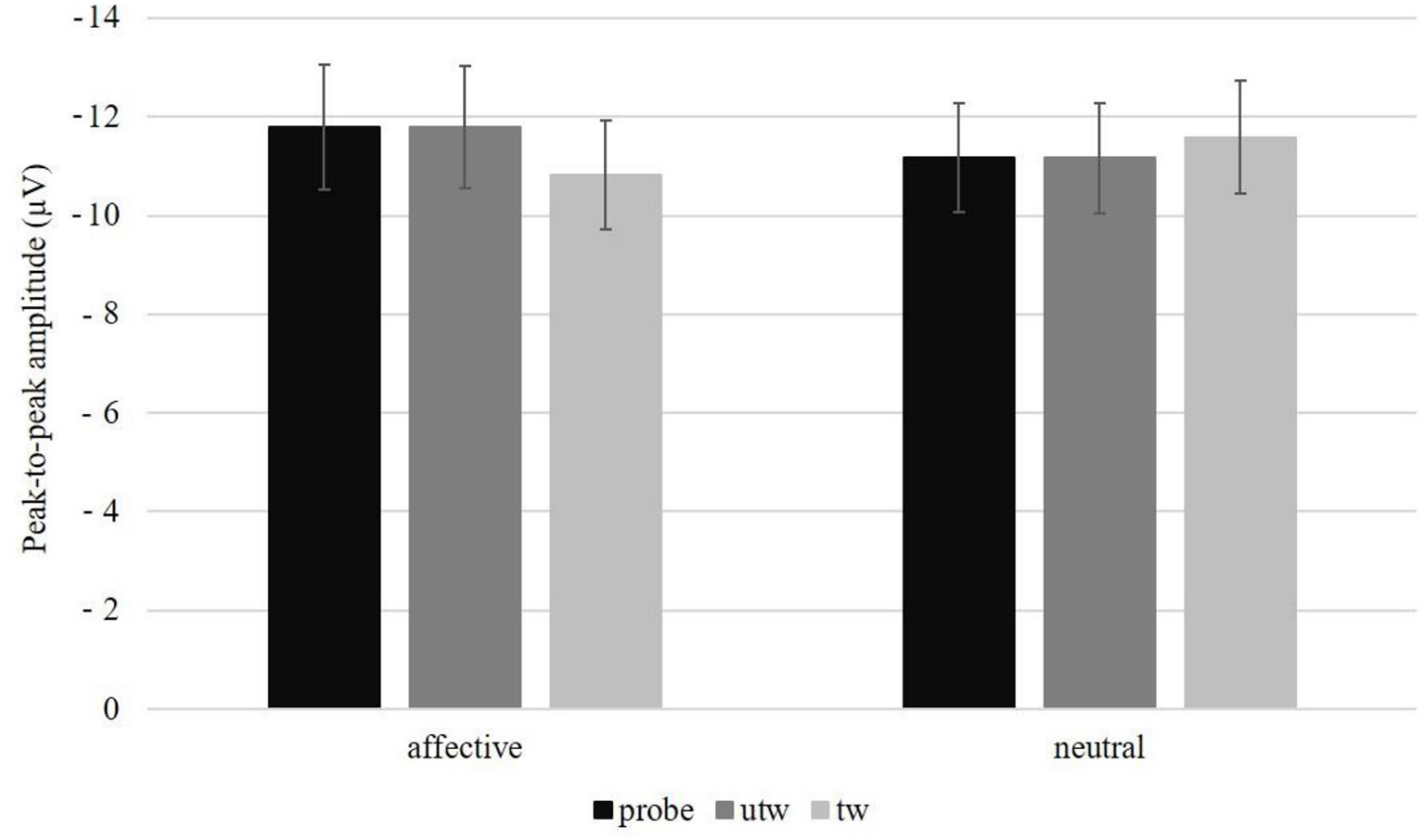
Peak-to-peak amplitude in microvolt (μV) and standard errors averaged over electrode positions (Fz, FCz, Cz, Pz) per picture type and conditions (affective vs. neutral). probe, untrustworthy-probe; utw, truthful untrustworthy; tw, truthful trustworthy.

A significant main effect of Position was observed, *F*_(3, 78)_ = 29.38, *p* < 0.01, η_*p*_^2^ = 0.53, ε = 0.46. Simple contrasts revealed a marginally more negative peak-to-peak N2 amplitude at FCz compared to Fz, *F*_(1, 26)_ = 3.43, *p* = 0.08, η_*p*_^2^ = 0.12, and a significantly more negative FCz amplitude compared to Cz, *F*_(1, 26)_ = 5.33, *p* = 0.03, η_*p*_^2^ = 0.17, and compared to Pz, *F*_(1, 26)_ = 38.79, *p* < 0.01, η_*p*_^2^ = 0.60. Furthermore, peak-to-peak N2 amplitudes were found to be more negative at Fz compared to Pz, *F*_(1, 26)_ = 26.63, *p* < 0.01, η_*p*_^2^ = 0.51 and at Cz compared to Pz, *F*_(1, 26)_ = 41.85, *p* < 0.01, η_*p*_^2^ = 0.62 (Table 2). In contrast to the other two quantifications, we found a significant interaction of Picture type × Condition, *F*_(2, 52)_ = 5.34, *p* = 0.01, η_*p*_^2^ = 0.17, ε = 0.96, but no Picture type main effect, *F*_(2, 52)_ = 1.16, *p* = 0.32, ε = 0.81. As indicated by the main effect of Picture type in the affective Condition, *F*_(2, 58)_ = 5.59, *p* = 0.01, η_*p*_^2^ = 0.16, ε = 0.99, the peak-to-peak N2 amplitude was significantly more negative following untrustworthy-probes compared to truthful trustworthy pictures, *F*_(1, 29)_ = 9.11, *p* = 0.01, η_*p*_^2^ = 0.24, and following truthful untrustworthy compared to truthful trustworthy pictures, *F*_(1, 29)_ = 7.47, *p* = 0.01, η_*p*_^2^ = 0.21, but not following untrustworthy-probes compared to truthful untrustworthy pictures, *F*_(1, 29)_ < 0.01, *p* = 0.96 (Figure 4). The Picture type main effect in the neutral Condition was not significant, *F*_(2, 58)_ = 1.26, *p* = 0.29, ε = 0.96. There were no interactions of Picture type × Task sequence, *F*_(2, 52)_ = 0.36, *p* = 0.66, ε = 0.81, or Picture type × Gender, *F*_(2, 52)_ = 1.48, *p* = 0.24, ε = 0.81 (for the results of a *post-hoc* exploration of all remaining main effects, two-way interactions and higher-order interactions see Supplementary Table 4).

### Comparison of Quantification Methods

The main effect of Position for the N2 amplitude was found to be robust across all quantification methods (mean amplitude, baseline-to-peak amplitude and peak-to-peak amplitude). Small effect sizes *r* were found for mean amplitudes (*r* = 0.19) and baseline-to-peak amplitudes (*r* = 0.21) whereas a moderate effect was found for the peak-to-peak amplitude (*r* = 0.49; Table 3). The Picture type main effect was only found to be robust across mean and baseline-to-peak amplitude. Small effect sizes *r* were observed for both quantification methods (mean amplitude: *r* = 0.10; baseline-to-peak: *r* = 0.05; Table 3). Thereby, the correlations between the trustworthiness ratings of the stimuli obtained from the current sample and the picture-wise N2 amplitudes are significant only in a few cases (see Supplementary Table 5). The expected Picture type × Condition interaction was only revealed using the peak-to-peak quantification. Thus, mean and baseline-to-peak amplitudes were appropriate to illustrate the expected Picture type main effect, whereas the peak-to-peak amplitude was particularly qualified to illustrate the Picture type × Condition interaction as predicted in hypothesis 2.

**Table 3:** Comparison of effect sizes between effects measured with mean, baseline-to-peak and peak-to-peak amplitude.

	**(1) mean amplitude**	**(2) baseline-to-peak amplitude**	**(3) peak-to-peak amplitude**	**Difference of effect sizes**
Main effect 1: Position	^*^ η_*p*_^2^ = 0.28 *r* = 0.19	^*^ η_*p*_^2^ = 0.30 *r* = 0.21	^*^ η_*p*_^2^ = 0.53 *r* = 0.49	*η_*p*_*^2^: 1–2: −0.02 2–3: −0.23 1–3: −0.25 *r*: 1–2: −0.02 2–3: −0.28 1ȓ3: −0.30
Main effect 2: Picture type	^*^ η_*p*_^2^ = 0.17 *r* = 0.10	^*^ η_*p*_^2^ = 0.10 *r* = 0.05		*η_*p*_*^2^: 1–2: 0.07 *r*: 1–2: 0.05
Interaction 1: Picture Type × Task Sequence		+ *η_*p*_*^2^ = 0.10 *r* = 0.05		
Interaction 2: Picture type × Condition			^*^ η_*p*_^2^ = 0.17 *r* = 0.10	

* *is significant. + is marginally significant. r = effect size r*.

### Behavioral Data

**Figure 5:**
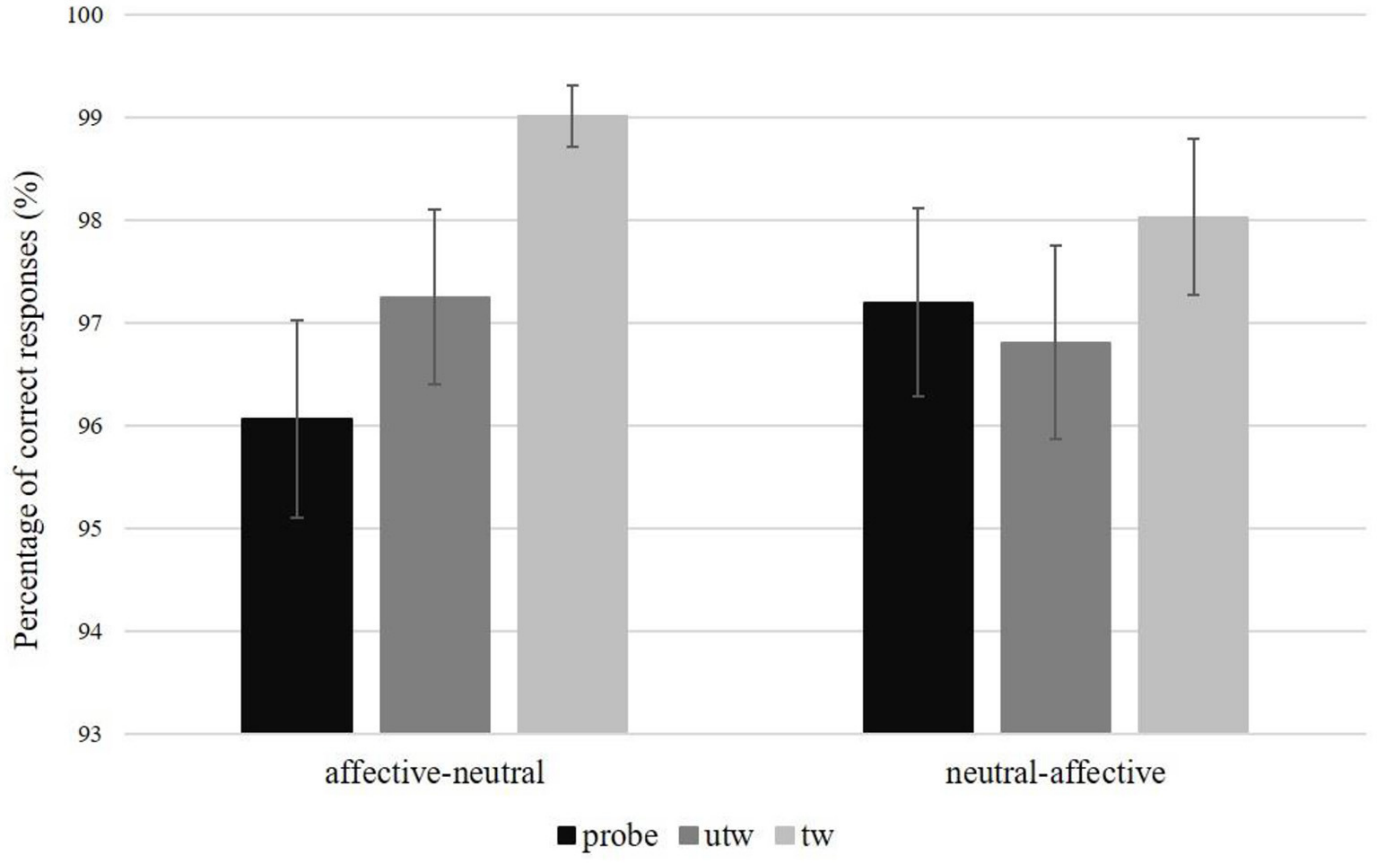
Percentage of correct responses and standard errors per picture type and task sequence. probe, untrustworthy-probe; utw, truthful untrustworthy; tw, truthful trustworthy.

For the percentage of correct responses there was a significant Picture type main effect, *F*_(2, 52)_ = 8.72, *p* < 0.01, η_*p*_^2^ = 0.25, ε = 0.91. Simple contrasts indicated a significantly higher correct rate (i.e., percentage of correct responses) for truthful trustworthy pictures compared to untrustworthy-probe pictures, *F*_(1, 26)_ = 14.70, *p* < 0.01, η_*p*_^2^ = 0.36, and for truthful trustworthy pictures compared to truthful untrustworthy pictures, *F*_(1, 26)_ = 7.87, *p* = 0.01, η_*p*_^2^ = 0.23 (Table 4; for an overview of percentage of correct responses and response times for the main and interaction effects of Picture type, Condition, and Task sequence, see Supplementary Tables 6–9). The correct rates of the latter two picture types did not differ significantly, *F*_(1, 26)_ = 1.07, *p* = 0.31. A marginally significant interaction of Picture type × Task sequence was found for the correct rate of responses, *F*_(2, 52)_ = 2.64, *p* = 0.09, η_*p*_^2^ = 0.09, ε = 0.91 (Figure 5). No significant interactions for correct rates were found for Picture type × Condition, *F*_(2, 52)_ = 0.90, *p* = 0.40, ε = 0.87, or Picture type × Gender, *F*_(2, 52)_ = 1.33, *p* = 0.27, ε = 0.91 (for the results of a *post-hoc* exploration of all remaining main effects, two-way interactions and higher-order interactions see Supplementary Table 9).

**Table 4:** Percentage of correct responses (%) and mean response times (ms) in correct trials.

**Picture type**	**Percentage of correct responses**	**Response times**
Untrustworthy-probe		
Overall	96.63 (3.60) [0.66]	353.88 (146.39) [26.73]
Affective	96.80 (5.11) [0.93]	347.70 (163.15) [29.79]
Neutral	96.47 (5.63) [1.03]	360.06 (161.52) [29.49]
Truthful untrustworthy		
Overall	97.03 (3.42) [0.62]	341.94 (151.84) [27.72]
Affective	97.73 (3.39) [0.62]	335.34 (156.63) [28.60]
Neutral	96.33 (5.29) [0.97]	348.55 (165.47) [30.21]
Truthful trustworthy		
Overall	98.52 (2.25) [0.41]	321.85 (135.75) [24.79]
Affective	99.33 (1.42) [0.26]	304.60 (126.95) [23.18]
Neutral	97.71 (4.02) [0.74]	339.10 (160.56) [29.31]

*Scores are presented in mean (standard deviation, SD) [standard error of the mean, SEM]*.

**Figure 6:**
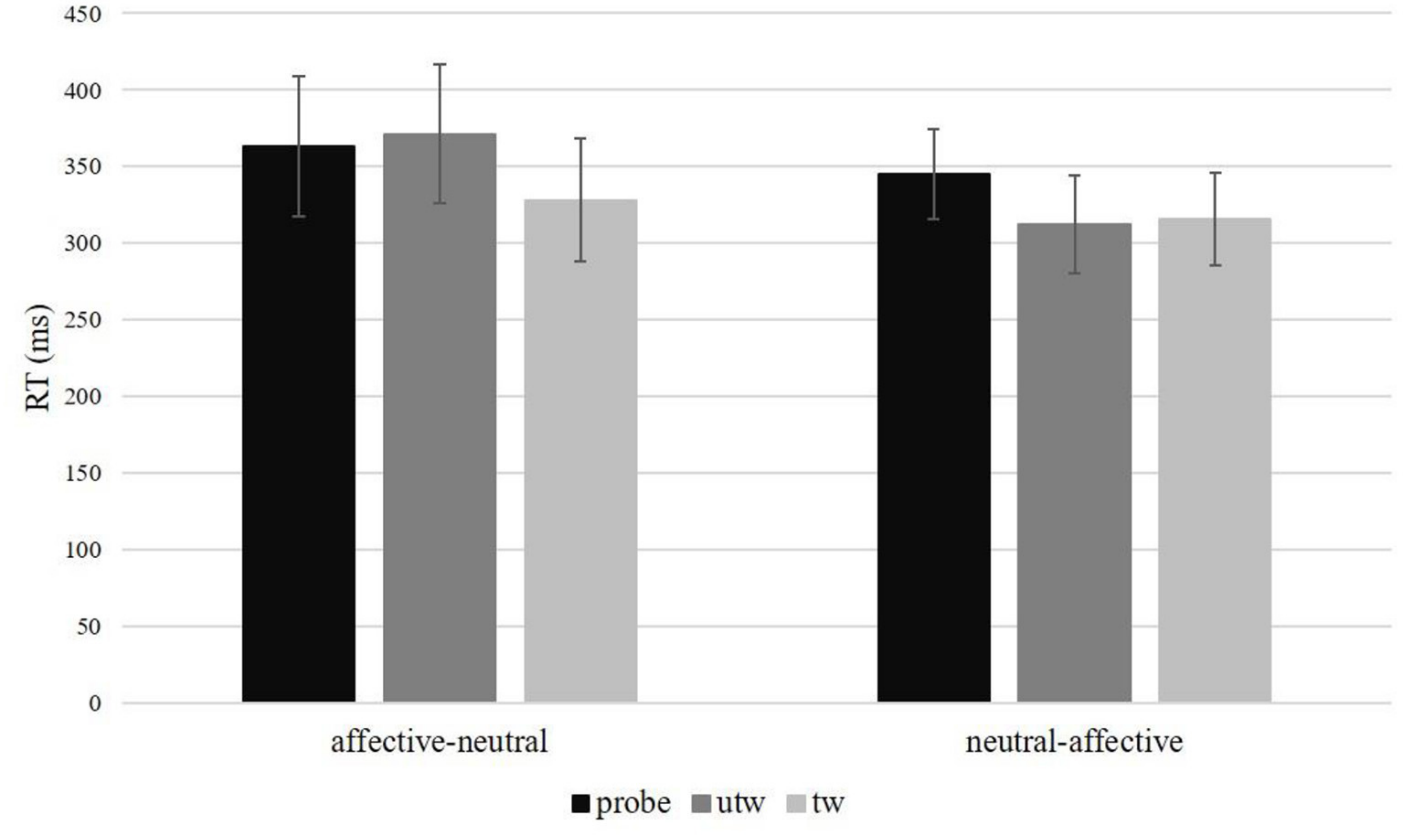
Response times in milliseconds (ms) and standard errors per picture type and task sequence. probe, untrustworthy-probe; utw, truthful untrustworthy; tw, truthful trustworthy.

There was a significant Picture type main effect of mean response times in correct trials, *F*_(2, 52)_ = 7.61, *p* < 0.01, η_*p*_^2^ = 0.23, ε = 0.85. Simple contrasts revealed significantly shorter response times to truthful trustworthy pictures compared to untrustworthy-probe pictures, *F*_(1, 26)_ = 13.05, *p* < 0.01, η_*p*_^2^ = 0.33, and in truthful trustworthy pictures compared to truthful untrustworthy pictures, *F*_(1, 26)_ = 4.68, *p* = 0.04, η_*p*_^2^ = 0.13 (Table 4). Response times for truthful untrustworthy pictures were only marginally shorter than for untrustworthy-probes, *F*_(1, 26)_ = 3.42, *p* = 0.08, η_*p*_^2^ = 0.12. A significant interaction of Picture type × Task sequence was found, *F*_(2, 52)_ = 4.53, *p* = 0.02, η_*p*_^2^ = 0.24, ε = 0.85. This interaction can be attributed to a main effect of Picture type in the Task sequence affective-neutral, *F*_(2, 28)_ = 11.06, *p* < 0.01, η_*p*_^2^ = 0.44, ε = 0.99, where significantly shorter response times were found for truthful trustworthy pictures compared to untrustworthy-probe, *F*_(1, 14)_ = 12.41, *p* < 0.01, η_*p*_^2^ = 0.47, and truthful untrustworthy pictures, *F*_(1, 14)_ = 19.53, *p* < 0.01, η_*p*_^2^ = 0.58, but not between untrustworthy-probe and truthful untrustworthy pictures, *F*_(1, 14)_ = 0.76, *p* = 0.40. The Picture type main effect in the Task sequence neutral-affective was only marginally significant, *F*_(2, 28)_ = 3.86, *p* = 0.05, η_*p*_^2^ = 0.22, ε = 0.71 (Figure 6). No significant effects regarding mean response times were found for Picture type × Condition, *F*_(2, 52)_ = 0.85, *p* = 0.42, η_*p*_^2^ = 0.03, ε = 0.84, or Picture type × Gender, *F*_(2, 52)_ = 0.03, *p* = 0.95, ε = 0.85 (for the results of a *post-hoc* exploration of all remaining main effects, two-way interactions and higher-order interactions see Supplementary Table 9).

## Discussion

In order to further understand the role of conflict monitoring in a social deception task, we investigated frontal N2 effects using an adapted CIT involving the social attribute (un)trustworthiness. Participants were instructed to either truthfully indicate a face's trustworthiness (truthful trustworthy), truthfully indicate a face's untrustworthiness (truthful untrustworthy), or conceal a face's untrustworthiness (untrustworthy-probe). We expected the N2 amplitude to be more negative following untrustworthy-probe stimuli compared to truthful trustworthy or truthful untrustworthy stimuli (hypothesis 1), as deception required increased conflict monitoring in previous deception studies (Wu et al., 2009; Fu et al., 2017). Furthermore, we hypothesized a larger frontal N2 amplitude following untrustworthy-probes compared to truthful trustworthy or truthful untrustworthy stimuli in the affective vs. neutral condition (hypothesis 2). Three quantification methods (mean, baseline-to-peak-, peak-to-peak amplitude) were compared, in order to identify the most applicable quantification method for the frontal N2 in an adapted CIT.

The main results of the study were the following: The typical N2 position effect (cf. Nieuwenhuis et al., 2003; Amodio et al., 2008; Folstein and van Petten, 2008) was found indicating a more pronounced N2 at fronto-central sites (FCz) compared to fronto (Fz) and parietal sites (Pz). Applying the mean or baseline-to-peak quantification, a Picture type main effect was found. According to hypothesis 1, a more negative frontal N2 amplitude was found following untrustworthy-probes compared to truthful trustworthy stimuli. This result is in line with the assumption that deceptive responses involve conflict monitoring (Wu et al., 2009; Fu et al., 2017). Unexpectedly, the frontal N2 amplitude was also more negative following truthful untrustworthy stimuli compared to truthful trustworthy stimuli and equally negative between truthful untrustworthy stimuli and untrustworthy-probes. This suggests that the frontal N2 amplitude is sensitive to affective evaluations of social attributes in a social deception task. Botvinick (2007) as well as Dreisbach and Fischer (2011) argued that aversive signals of various kinds (e.g., response-conflict, negative feedback, pain, social rejection) can trigger compensatory shifts in control and either mobilize effort or lead to task avoidance. In the current study, the aversive evaluation of the social attribute untrustworthiness might have served as an aversive signal itself even in the absence of response conflict. Since task avoidance was not an option, cognitive effort was increased by an activation of the ACC, illustrated by a more negative N2 amplitude (Dreisbach and Fischer, 2011). Another possible explanation is the occurrence of an increased conflict following truthful untrustworthy pictures between the action tendencies to assign these pictures to the truthful untrustworthy or untrustworthy-probe type before even considering a deceptive response (Botvinick et al., 2001; Botvinick, 2007). In addition to a 1:1:1 picture type ratio, two-thirds of the stimuli should be responded to with “trustworthy.” Thus, the truthful untrustworthy picture type differs from the other two picture types in response frequency. The anticipated rareness of the “untrustworthy” response vs. the more frequent “trustworthy” responses might be a source of the conflict that is expressed in an increased N2 amplitude following truthful untrustworthy pictures. However, previous N2 effects for example in go/nogo tasks referred to the stimulus ratio and not to an anticipated response ratio (Leue et al., 2012b). Thus, effects of varying response ratios on the N2-related conflict monitoring intensity in deception tasks have not yet been studied. To investigate the influence of response frequency, future studies could also implement a trustworthy-probe picture type and use other paradigms such as the Differentiation of Deception Paradigm (Furedy et al., 1988) that has been used in previous studies, which reported increased conflict monitoring (in terms of a more negative N2) during deception (Hu et al., 2011; Suchotzki et al., 2015; but see Pfister et al., 2014). All N2 findings on picture type were independent of the participants' gender and task sequence.

Addressing hypothesis 2, a more negative N2 peak-to-peak amplitude was found following untrustworthy-probes compared to truthful trustworthy pictures and also following truthful untrustworthy compared to truthful trustworthy pictures but not following untrustworthy-probes compared to truthful untrustworthy pictures in the affective vs. neutral condition. This suggests that the amount of conflict monitoring not only increases as the subject's conviction of the faces untrustworthiness that has to be concealed increases, but also as truthful untrustworthy pictures appeared. As described earlier, two thirds of the stimuli should be responded to with “trustworthy” and only one third with “untrustworthy.” The rarity of the to-be-prepared “untrustworthy” response may have led to a default tendency to prepare a “trustworthy” response. It is possible that the need to prepare a rare “untrustworthy” response may have resulted in a conflict with this default tendency, expressed by an increased N2. In this respect, we cannot exclude that the increased N2 following untrustworthy stimuli reflects more than one cognitive process that is related to conflict monitoring. These cognitive processes could be disentangled in future studies using principal component analysis (Sun et al., 2011). Applying the mean or baseline-to-peak quantification, hypothesis 2 could not be confirmed for the Picture type × Condition interaction. Although Luck (2014) argues that peak-to-peak amplitudes in the sense of difference waves might have a worse signal-to-noise ratio, a higher number of artifact-free epochs (here at least 25 artifact-free epochs) might, however, improve the signal-to-noise ratio in a deception task (cf. Kleene et al., 2021 for reliability of the N2 in another deception task). This might explain why the Picture type × Condition interaction was particularly observed for the peak-to-peak amplitude. Moreover, we did not investigate difference waves of the experimental conditions but inserted each experimental condition in the statistical analysis.

The mean and baseline-to-peak N2 amplitudes were found to cover the Picture type × Condition interaction, whereas the peak-to-peak quantification as one option to disentangle ERP components highlights conflict monitoring intensity during deception especially following faces that were rated more affectively untrustworthy (Dignath et al., 2020). These results suggest that the selection of the quantification method should be part of a hypothesis-related investigation of the N2 amplitude in an adapted social deception task. More specifically, mean and baseline-to-peak amplitudes are superior for examining the main effect of picture type, whereas the peak-to-peak amplitude should be used to investigate the differentiation of the extent of conflict monitoring between conditions. To which other hypotheses the findings on quantification methods can be extended should be investigated in future studies.

Analyses of the behavioral data revealed lower correct rates following deceptive stimuli (untrustworthy-probe) compared to truthful trustworthy stimuli. However, correct rates did not differ between untrustworthy-probe and truthful untrustworthy stimuli. Response times were significantly shorter following truthful trustworthy compared to untrustworthy-probe and truthful untrustworthy stimuli, but did not differ between the latter two. There was only one category of trustworthy faces (truthful trustworthy) but two categories of untrustworthy faces (untrustworthy-probe and truthful untrustworthy). Thus, the higher conflict monitoring intensity to untrustworthy compared to trustworthy stimuli might be either due to more cognitive demand or more aversiveness of the anticipated “untrustworthy” response (Leue et al., 2012a, 2014). Moreover, the truthful untrustworthy stimuli were the only category to be responded to with “untrustworthy.” Thus, a comparatively slower response time following truthful untrustworthy stimuli may also have resulted from the rareness of the response.

### Limitations and Future Directions

It should be mentioned that the current study examined instructed deception. Although it has been demonstrated that both, voluntary (e.g., self-chosen deceptive stimuli) and instructed deception involve conflict monitoring (e.g., Hu et al., 2011, 2015; Leue and Beauducel, 2015), it would be interesting to further investigate the role of the intention to deceive, especially in social deception situations. Accordingly, Sai et al. (2018) stated that it is the deceptive intention that elicits conflict in a social deception situation, not the false content of a deceptive response.

As mentioned in the Methods, the assignment of trustworthiness to the response keys and the assignment of the neutral pictures to the picture types were constant across participants (cf. Koeckritz et al., 2019). To avoid any potential influences of key selection and picture assignment in the neutral condition, both assignments should be counterbalanced in further studies. Furthermore, a comparison of P3 findings based on Koeckritz et al.'s (2019) trustworthiness condition and the present trustworthiness condition would be worthwhile in future research.

A major limitation of the current study was the reduced sample size that did not allow for analysis of personality traits that may affect the neurocognitive processes during deception. Therefore, further studies are needed to investigate the role of conflict monitoring during deception depending on personality traits such as expression of the behavioral inhibition system (BIS). Since it can be assumed that deception is based on an interplay of several complex neurocognitive processes, further research could consider additional processes and broaden the focus to include not only stimulus-locked but also response-locked ERPs. One promising process that should be studied in future in the context of deception with social attributes is cognitive control, which also involves the ACC and is reflected by the ERN/Ne (Johnson et al., 2004, 2008; Leue et al., 2012a). More information about the role of cognitive control in deception about social attributes would not only further our understanding of the neurocognitive basis of deception but may also improve the detection of truthful and deceptive responses.

## Data Availability Statement

The raw data supporting the conclusions of this article will be made available by the authors, without undue reservation.

## Ethics Statement

The studies involving human participants were reviewed and approved by Ethics Committee of the Medical Faculty of Kiel University, Germany. The patients/participants provided their written informed consent to participate in this study.

## Author Contributions

F-EH and AL contributed to the study conceptualization. F-EH collected and prepared data, performed statistical analyses, wrote the first draft of the manuscript, and revised the draft based on feedback of AL. Both authors read and approved the submitted version of the manuscript.

## Conflict of Interest

The authors declare that the research was conducted in the absence of any commercial or financial relationships that could be construed as a potential conflict of interest.

## Publisher's Note

All claims expressed in this article are solely those of the authors and do not necessarily represent those of their affiliated organizations, or those of the publisher, the editors and the reviewers. Any product that may be evaluated in this article, or claim that may be made by its manufacturer, is not guaranteed or endorsed by the publisher.
