# Supplementary material for: Concealing Untrustworthiness: The Role of Conflict Monitoring in a Social Deception Task
Source: Front Psychol. 2021 Aug 20;12:718334. doi: 10.3389/fpsyg.2021.718334 (PMC8417705; doi:10.3389/fpsyg.2021.718334)
Supplement: Supplementary file 1 [file Data_Sheet_1.pdf]

(A)

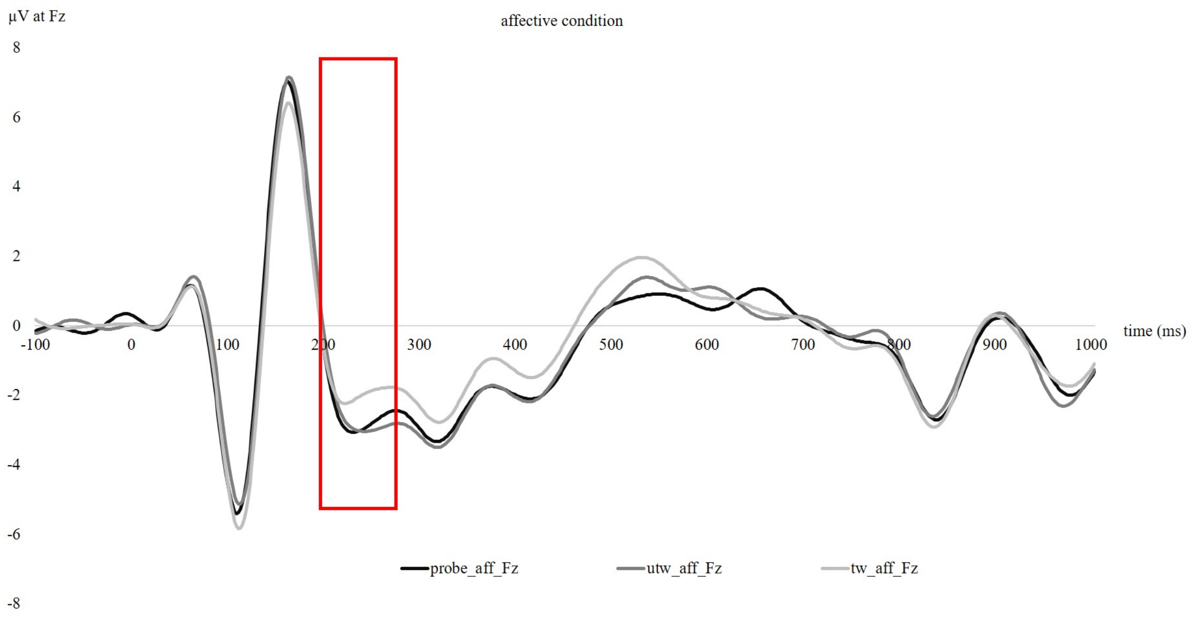

(B)

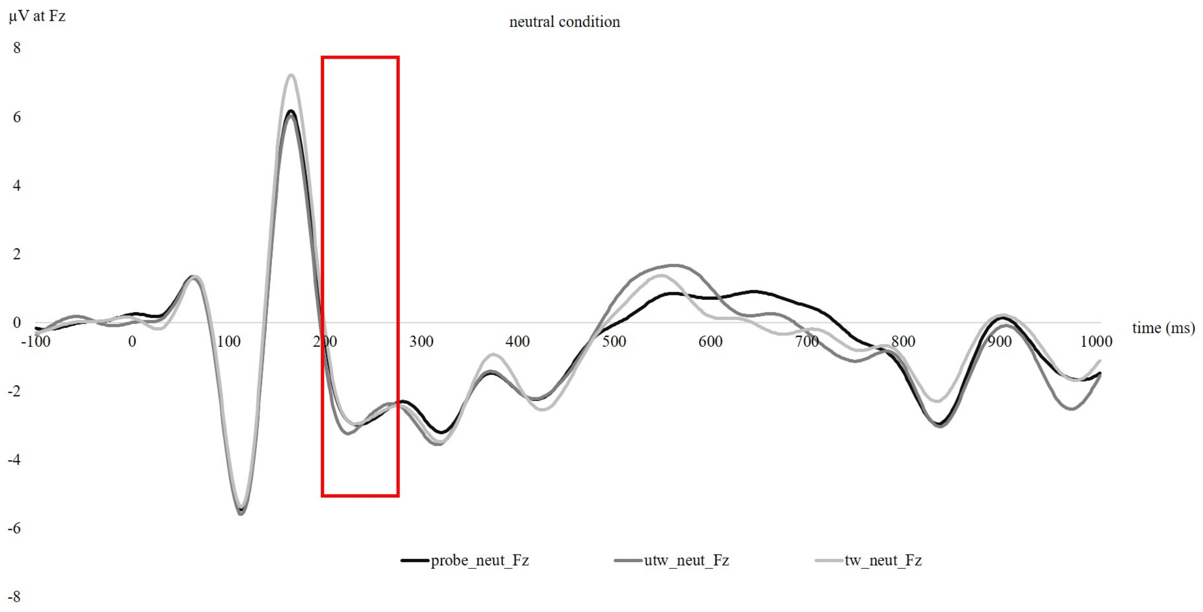

Figure S1. Stimulus-locked grand averages at Fz from 0 to 1000 ms post-stimulus, with a 100 ms pre-stimulus baseline for three picture types (untrustworthy-probe, truthful untrustworthy, truthful trustworthy) in the affective condition (A) and neutral condition (B). Stimulus was presented from 0 to 700 ms. N2 amplitudes were identified between 200 and 275 ms post-stimulus.

(A)

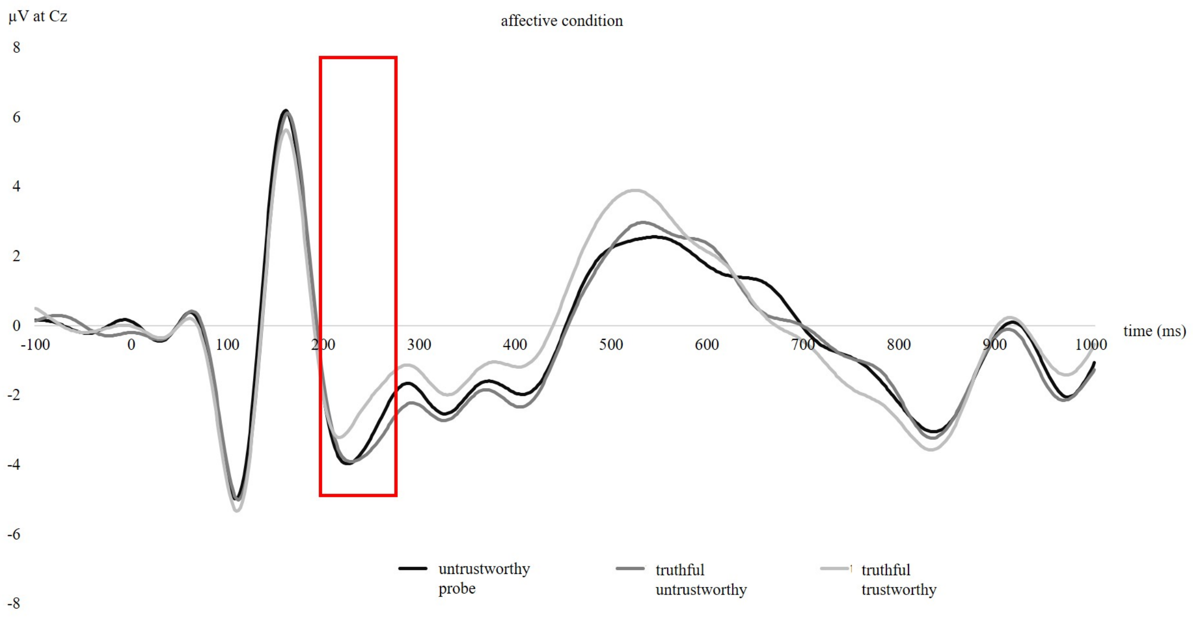

(B)

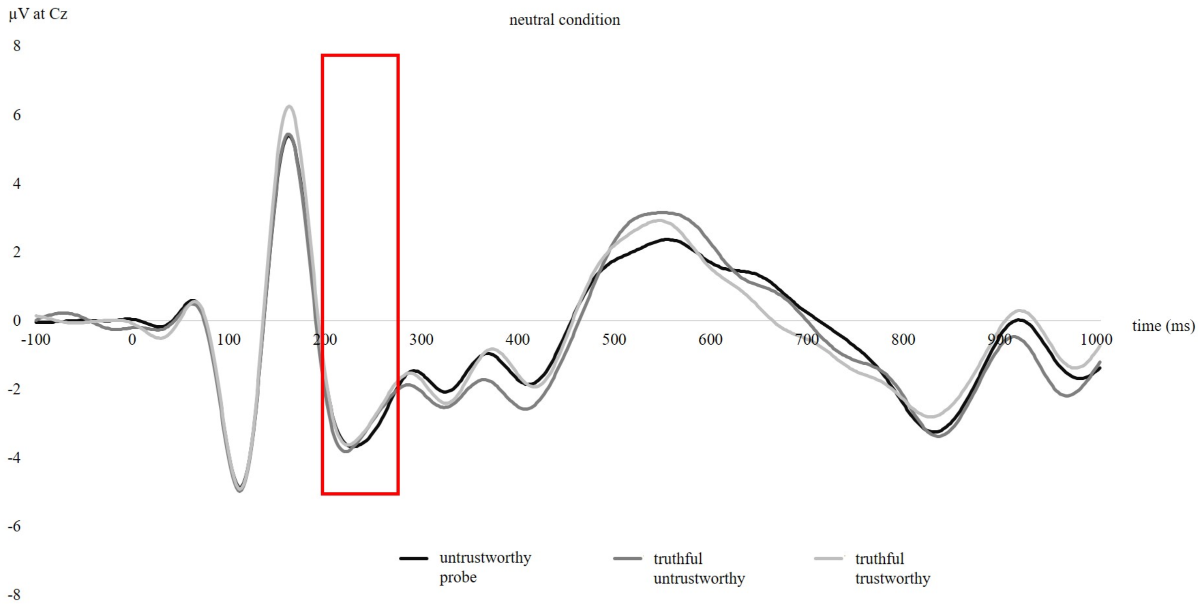

Figure S2. Stimulus-locked grand averages at Cz from 0 to 1000 ms post-stimulus, with a 100 ms pre-stimulus baseline for three picture types (untrustworthy-probe, truthful untrustworthy, truthful trustworthy) in the affective condition (A) and neutral condition (B). Stimulus was presented from 0 to 700 ms. N2 amplitudes were identified between 200 and 275 ms post-stimulus.

(A)

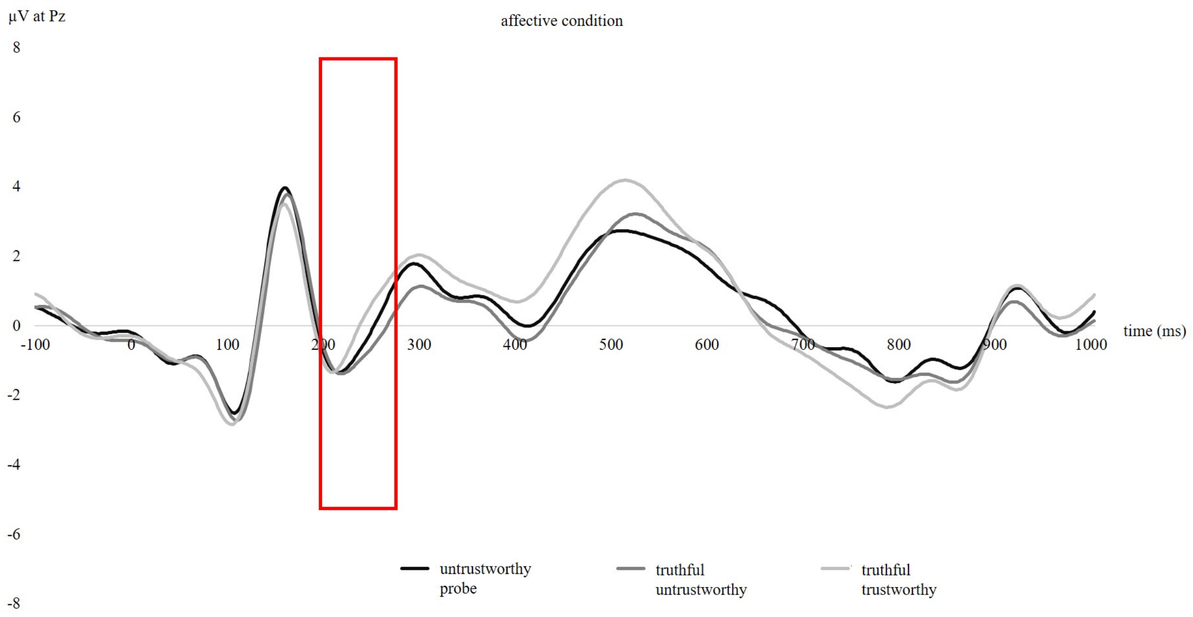

(B)

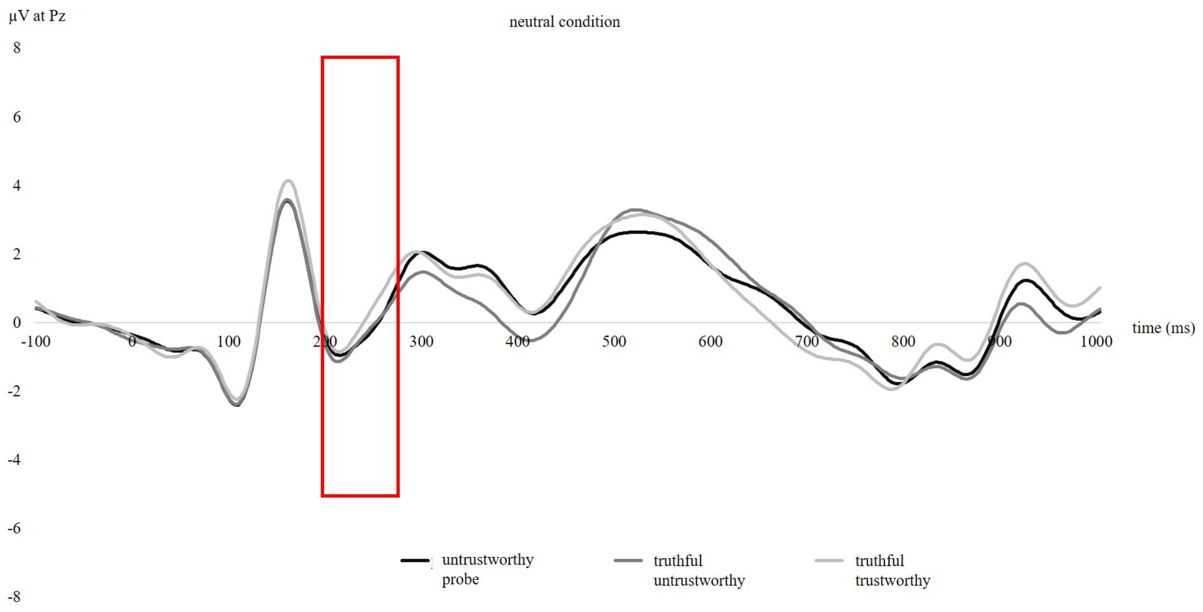

Figure S3. Stimulus-locked grand averages at Pz from 0 to 1000 ms post-stimulus, with a 100 ms pre-stimulus baseline for three picture types (untrustworthy-probe, truthful untrustworthy, truthful trustworthy) in the affective condition (A) and neutral condition (B). Stimulus was presented from 0 to 700 ms. N2 amplitudes were identified between 200 and 275 ms post-stimulus.

Table S1. N2 amplitudes in microvolt (µV) for Electrode position, Picture type, Condition, Task sequence and Gender of the participant based on different quantifications.

	Mean amplitude	Baseline-to-Peak amplitude	Peak-to-Peak amplitude N2-P2
Electrode position[1,2,3,4]	*	*	*
Fz	-2.43 (3.56) [0.65]	-4.71 (3.86) [0.70]	-12.57 (6.58) [1.20]
FCz	-3.00 (3.43) [0.63]	-5.27 (3.69) [0.67]	-13.08 (6.94) [1.27]
Cz	-2.95 (3.35) [0.61]	-5.31 (3.55) [0.65]	-12.40 (7.18) [1.32]
Pz	-0.26 (3.83) [0.70]	-2.58 (3.59) [0.66]	-7.50 (5.74) [1.05]
Picture type[2,3,4,5]	*	*	
Probe	-3.13 (3.38) [0.62]	-5.45 (3.65) [0.67]	-13.19 (7.07) [1.29]
Utw	-3.25 (3.72) [0.68]	-5.42 (4.07) [0.74]	-13.18 (7.14) [1.30]
Tw	-2.64 (3.44) [0.63]	-4.95 (3.60) [0.66]	-12.87 (6.76) [1.23]
Condition[1,3,4,5]			
Affective	-2.95 (3.48) [0.64]	-5.18 (3.68) [0.67]	-13.12 (7.21) [1.32]
Neutral	-3.05 (3.52) [0.64]	-5.37 (3.82) [0.70]	-13.04 (6.80) [1.24]
Task sequence[1,2,4,5]			
Affective-neutral	-2.78 (3.98) [1.03]	-5.12 (4.02) [1.04]	-14.52 (7.15) [1.85]
Neutral-affective	-3.23 (2.90) [0.75]	-5.43 (3.46) [0.89]	-11.65 (6.66) [1.72]
Gender of participant[1,2,3,5]			
Male	-3.00 (4.01) [1.04]	-5.23 (4.51) [1.16]	-13.55 (8.57) [2.21]
Female	-3.01 (2.87) [0.74]	-5.32 (2.81) [0.72]	-12.61 (5.10) [1.32]

Note. Scores are presented in mean (standard deviation, SD) [standard error of the mean, SEM]. * Significant at $\alpha = .05$. [1] Amplitudes are averaged over picture types. [2] Amplitudes are averaged over conditions (affective vs. neutral). [3] Amplitudes are averaged over task sequence (affective-neutral vs. neutral-affective). [4] Amplitudes are averaged over gender of the participant. [5] Amplitudes are measured at FCz. Probe = Untrustworthy-probe. Utw = Truthful untrustworthy. Tw = Truthful trustworthy.

Table S2. N2 amplitudes in microvolt (µV) measured at FCz for interactions of Picture type, Condition, Task sequence and Gender of the participant based on different quantifications.

	Mean amplitude	Baseline-to-Peak amplitude	Peak-to-Peak amplitude N2-P2
Picture type × Condition[1,2]			*
Probe aff.	-3.17 (3.66) [0.67]	-5.40 (3.91) [0.71]	-13.53 (7.70) [1.41]
Utw aff.	-3.34 (3.84) [0.70]	-5.38 (4.13) [0.75]	-13.54 (7.61) [1.39]
Tw aff.	-2.35 (3.44) [0.63]	-4.76 (3.60) [0.66]	-12.29 (6.67) [1.22]
Probe neut.	-3.09 (3.47) [0.63]	-5.51 (3.71) [0.68]	-12.85 (6.77) [1.24]
Utw neut.	-3.15 (3.83) [0.70]	-5.46 (4.32) [0.79]	-12.83 (6.94) [1.27]
Tw neut.	-2.92 (3.67) [0.67]	-5.15 (3.86) [0.71]	-13.45 (6.97) [1.27]
Picture type × Task sequence[2,3]		+	
Probe aff.-neut.	-3.14 (3.75) [0.97]	-5.62 (3.79) [0.98]	-14.62 (7.27) [1.88]
Utw aff.-neut.	-2.95 (4.38) [1.13]	-5.26 (4.58) [1.18]	-14.77 (7.32) [1.89]
Tw aff.-neut.	-2.24 (3.99) [1.03]	-4.49 (3.95) [1.02]	-14.57 (7.29) [1.87]
Probe neut.-aff.	-3.12 (3.09) [0.80]	-5.29 (3.63) [0.94]	-11.76 (6.81) [1.76]
Utw neut.-aff.	-3.54 (3.06) [0.79]	-5.58 (3.65) [0.94]	-11.60 (6.83) [1.76]
Tw neut.-aff.	-3.04 (2.87) [0.74]	-5.41 (3.29) [0.85]	-11.58 (6.82) [1.76]
Picture type × Gender[1,3]			
Probe male	-2.98 (3.78) [0.98]	-5.30 (4.27) [1.10]	-13.69 (8.73) [2.25]
Utw male	-3.32 (4.59) [1.18]	-5.47 (5.14) [1.33]	-13.87 (8.97) [2.31]
Tw male	-2.70 (3.85) [0.99]	-4.93 (4.26) [1.10]	-13.87 (8.97) [2.31]
Probe female	-3.28 (3.04) [0.79]	-5.61 (3.05) [0.79]	-12.69 (5.18) [1.34]
Utw female	-3.17 (2.77) [0.71]	-5.37 (2.82) [0.73]	-12.49 (4.92) [1.27]
Tw female	-2.58 (3.11) [0.80]	-4.97 (2.95) [0.76]	-12.45 (4.90) [1.27]
Condition × Task sequence[2,4]			
Aff. aff.-neut.	-2.77 (4.12) [1.06]	-5.07 (4.03) [1.04]	-14.73 (7.56) [1.95]
Neut. aff.-neut.	-2.78 (3.96) [1.02]	-5.17 (4.13) [1.07]	-14.30 (6.87) [1.77]
Aff. neut.-aff.	-3.14 (2.84) [0.73]	-5.29 (3.42) [0.88]	-11.51 (6.71) [1.73]
Neut. neut.-aff.	-3.33 (3.13) [0.81]	-5.57 (3.62) [0.93]	-11.78 (6.72) [1.73]
Condition × Gender[1,4]			
Aff. male	-3.00 (4.03) [1.04]	-5.22 (4.46) [1.15]	-13.62 (8.93) [2.31]
Neut. male	-3.00 (4.12) [1.06]	-5.25 (4.66) [1.20]	-13.48 (8.32) [2.15]
Aff. female	-2.91 (2.98) [0.77]	-5.14 (2.85) [0.74]	-12.62 (5.24) [1.35]
Neut. female	-3.11 (2.95) [0.76]	-5.49 (2.91) [0.75]	-12.61 (5.11) [1.32]
Task sequence × Gender[3,4]			
Aff.-neut. male	-2.46 (4.88) [1.73]	-4.40 (5.06) [1.79]	-14.43 (9.18) [3.24]
Neut.-aff. male	-3.62 (2.99) [1.13]	-6.19 (3.95) [1.49]	-12.54 (8.41) [3.18]
Aff.-neut. female	-3.14 (2.97) [1.12]	-5.95 (2.53) [0.96]	-14.61 (4.59) [1.73]
Neut.-aff. female	-2.89 (2.99) [1.06]	-4.76 (3.09) [1.09]	-10.87 (5.15) [1.82]

Note. Scores are presented in mean (standard deviation, SD) [standard error of the mean, SEM]. * Significant at $\alpha = .05$. + marginally significant at $\alpha = .10$. [1] Amplitudes are averaged over task sequence (affective-neutral vs. neutral-affective). [2] Amplitudes are averaged over gender. [3] Amplitudes are averaged over conditions (affective vs. neutral). [4] Amplitudes are averaged over picture types. Probe = Untrustworthy-probe. Utw = Truthful untrustworthy. Tw = Truthful trustworthy. Aff. = affective. Neut. = neutral.

Table S3A. N2 amplitudes in microvolt (μV) measured at FCz for higher-order interactions of Picture type, Condition, Task sequence and Gender of the participant based on different quantifications.

	Mean amplitude	Baseline-to-Peak amplitude	Peak-to-Peak amplitude N2-P2
Picture type × Condition × Task sequence[1]			
Probe aff. aff.-neut.	-3.48 (4.09) [1.06]	-5.86 (4.01) [1.03]	-15.27 (8.01) [2.07]
Utw aff. aff.-neut.	-3.03 (4.56) [1.18]	-5.38 (4.73) [1.22]	-15.63 (7.99) [2.06]
Tw aff. aff.-neut.	-1.81 (4.08) [1.05]	-3.98 (3.86) [1.00]	-13.29 (7.15) [1.85]
Probe neut. aff.-neut.	-2.80 (3.84) [0.99]	-5.38 (3.87) [1.00]	-13.97 (6.91) [1.78]
Utw neut. aff.-neut.	-2.87 (4.42) [1.14]	-5.14 (4.68) [1.21]	-13.90 (6.86) [1.77]
Tw neut. aff.-neut.	-2.67 (4.06) [1.05]	-5.00 (4.27) [1.10]	-15.03 (7.13) [1.84]
Probe aff. neut.-aff.	-2.86 (3.29) [0.85]	-4.95 (3.89) [1.00]	-11.79 (7.23) [1.87]
Utw aff. neut.-aff.	-3.66 (3.08) [0.80]	-5.37 (3.61) [0.93]	-11.45 (6.85) [1.77]
Tw aff. neut.-aff.	-2.89 (2.68) [0.69]	-5.54 (3.27) [0.85]	-11.29 (6.25) [1.61]
Probe neut. neut.-aff.	-3.38 (3.16) [0.82]	-5.63 (3.67) [0.95]	-11.73 (6.67) [1.72]
Utw neut. neut.-aff.	-3.42 (3.27) [0.84]	-5.78 (4.06) [1.05]	-11.75 (7.09) [1.83]
Tw neut. neut.-aff.	-3.18 (3.36) [0.87]	-5.29 (3.56) [0.92]	-11.87 (6.66) [1.72]
Picture type × Condition × Gender[2]			
Probe aff. male	-3.09 (4.24) [1.09]	-5.30 (4.72) [1.22]	-14.03 (9.47) [2.45]
Utw aff. male	-3.49 (4.67) [1.21]	-5.48 (5.26) [1.36]	-14.12 (9.81) [2.53]
Tw aff. male	-2.41 (3.61) [0.93]	-4.86 (3.96) [1.02]	-12.71 (7.84) [2.02]
Probe neut. male	-2.86 (3.83) [0.99]	-5.29 (4.27) [1.10]	-13.34 (8.34) [2.15]
Utw neut. male	-3.15 (4.69) [1.21]	-5.46 (5.39) [1.39]	-13.63 (8.36) [2.16]
Tw neut. male	-2.99 (4.23) [1.09]	-5.00 (4.72) [1.22]	-13.47 (8.42) [2.17]
Probe aff. female	-3.25 (3.13) [0.81]	-5.50 (3.05) [0.79]	-13.02 (5.71) [1.47]
Utw aff. female	-3.20 (2.94) [0.76]	-5.27 (2.78) [0.72]	-12.96 (4.80) [1.24]
Tw aff. female	-2.30 (3.38) [0.87]	-4.65 (3.34) [0.86]	-11.87 (5.52) [1.42]
Probe neut. female	-3.32 (3.18) [0.82]	-5.72 (3.18) [0.82]	-12.36 (4.99) [1.29]
Utw neut. female	-3.14 (2.90) [0.75]	-5.46 (3.09) [0.80]	-12.03 (5.34) [1.38]
Tw neut. female	-2.86 (3.16) [0.82]	-5.29 (2.93) [0.76]	-13.43 (5.45) [1.41]
Condition × Task sequence × Gender[3]	*		
Aff. aff.-neut. male	-2.76 (4.95) [1.75]	-4.64 (5.00) [1.77]	-14.85 (9.52) [3.37]
Neut. aff.-neut. male	-2.16 (4.88) [1.73]	-4.15 (5.17) [1.83]	-14.01 (8.91) [3.15]
Aff. neut.-aff. male	-3.27 (3.02) [1.14]	-5.87 (4.03) [1.52]	-12.21 (8.71) [3.29]
Neut. neut.-aff. male	-3.97 (3.12) [1.18]	-6.51 (4.01) [1.52]	-12.87 (8.25) [3.12]
Aff. aff.-neut. female	-2.79 (3.33) [1.26]	-5.56 (2.87) [1.09]	-14.59 (5.24) [1.98]
Neut. aff.-neut. female	-3.49 (2.77) [1.05]	-5.34 (2.37) [0.89]	-14.63 (4.15) [1.57]
Aff. neut.-aff. female	-3.02 (2.87) [1.01]	-4.78 (1.98) [1.05]	-10.90 (4.91) [1.73]
Neut. neut.-aff. female	-2.77 (3.25) [1.15]	-4.75 (3.28) [1.16]	-10.83 (5.45) [1.93]

Note. Scores are presented in mean (standard deviation, SD) [standard error of the mean, SEM]. * Significant at $\alpha = .05$. [1] Amplitudes are averaged over gender. [2] Amplitudes are averaged over task sequence (affective-neutral vs. neutral-affective). [3] Amplitudes are averaged picture types. Probe = Untrustworthy-probe. Utw = Truthful untrustworthy. Tw = Truthful trustworthy. Aff. = affective. Neut. = neutral. For statistical results and additional significant higher-order interactions revealed by post-hoc explorations, see Table S4.

Table S3B. N2 amplitudes in microvolt (µV) measured at FCz for higher-order interactions of Picture type, Condition, Task sequence and Gender of the participant based on different quantifications.

	Mean amplitude	Baseline-to-Peak amplitude	Peak-to-Peak amplitude N2-P2
Picture type × Task sequence × Gender[1]			
Probe. aff.-neut. male	-2.68 (4.36) [1.54]	-4.76 (4.49) [1.59]	-14.62 (9.18) [3.35]
Utw. aff.-neut. male	-2.59 (5.65) [2.00]	-4.48 (5.98) [2.11]	-14.85 (9.77) [3.46]
Tw aff.-neut. male	-2.11 (4.69) [1.66]	-3.95 (4.76) [1.68]	-14.64 (9.75) [3.45]
Probe neut.-aff. male	-3.32 (3.30) [1.25]	-5.92 (4.27) [1.61]	-12.62 (8.78) [3.32]
Utw. neut.-aff. male	-4.16 (3.22) [1.22]	-6.60 (4.14) [1.57]	-12.75 (8.56) [3.24]
Tw neut.-aff. male	-3.38 (2.81) [1.06]	-6.05 (3.64) [1.38]	-12.69 (8.54) [3.22]
Probe aff.-neut. female	-3.67 (3.17) [1.20]	-6.60 (2.81) [1.06]	-14.61 (5.00) [1.89]
Utw. aff.-neut. female	-3.36 (2.68) [1.01]	-6.15 (2.36) [0.89]	-14.67 (3.66) [1.38]
Tw. aff.-neut. female	-2.39 (3.39) [1.28]	-5.10 (3.03) [1.15]	-14.66 (3.64) [1.37]
Probe neut.-aff. female	-2.95 (3.10) [1.10]	-4.74 (3.16) [1.12]	-11.01 (5.02) [1.78]
Utw neut.-aff. female	-3.00 (3.01) [1.06]	-4.68 (3.17) [1.12]	-10.59 (5.29) [1.87]
Tw neut.-aff. female	-2.74 (3.07) [1.09]	-4.86 (3.09) [1.09]	-10.64 (5.31) [1.88]
Picture type × Condition × Task sequence × Gender			
Probe aff. aff.-neut. male	-3.23 (4.83) [1.71]	-5.22 (4.81) [1.70]	-15.46 (9.99) [3.53]
Utw aff. aff.-neut. male	-3.02 (5.78) [2.04]	-4.91 (6.24) [2.20]	-15.80 (10.64) [3.76]
Tw aff. aff.-neut. male	-2.03 (4.60) [1.63]	-3.80 (4.35) [1.54]	-13.29 (8.36) [2.95]
Probe neut. aff.-neut. male	-2.12 (4.39) [1.55]	-4.29 (4.54) [1.60]	-13.78 (8.72) [3.08]
Utw neut. aff.-neut. male	-2.16 (5.67) [2.01]	-4.06 (5.94) [2.10]	-13.90 (9.09) [3.21]
Tw neut. aff.-neut. male	-2.19 (4.86) [1.72]	-4.10 (5.31) [1.88]	-14.35 (9.06) [3.20]
Probe aff. neut.-aff. male	-2.93 (3.81) [1.44]	-5.40 (5.00) [1.89]	-12.40 (9.33) [3.53]
Utw aff. neut.-aff. male	-4.03 (3.36) [1.27]	-6.14 (4.26) [1.61]	-12.19 (9.19) [3.47]
Tw aff. neut.-aff. male	-2.84 (2.30) [0.87]	-6.07 (3.38) [1.28]	-12.04 (7.81) [2.95]
Probe neut. neut.-aff. male	-3.70 (3.20) [1.21]	-6.43 (3.97) [1.50]	-12.84 (8.54) [3.23]
Utw neut. neut.-aff. male	-4.28 (3.31) [1.25]	-7.07 (4.58) [1.73]	-13.31 (8.16) [3.09]
Tw neut. neut.-aff. male	-3.91 (3.51) [1.33]	-6.03 (4.10) [1.55]	-12.46 (8.21) [3.10]
Probe aff. aff.-neut. female	-3.77 (3.41) [1.29]	-6.58 (3.06) [1.16]	-15.04 (5.77) [2.18]
Utw aff. aff.-neut. female	-3.04 (3.08) [1.16]	-5.92 (2.50) [0.94]	-15.43 (4.09) [1.54]
Tw aff. aff.-neut. female	-1.56 (3.75) [1.42]	-4.17 (3.55) [1.34]	-13.28 (6.14) [2.32]
Probe neut. aff.-neut. fem.	-3.56 (3.27) [1.23]	-6.62 (2.75) [1.04]	-14.19 (4.75) [1.80]
Utw neut. aff.-neut. female	-3.69 (2.56) [0.97]	-6.38 (2.59) [0.98]	-13.90 (3.66) [1.38]
Tw neut. aff.-neut. female	-3.22 (3.18) [1.20]	-6.03 (2.70) [1.02]	-15.81 (4.64) [1.76]
Probe aff. neut.-aff. female	-2.79 (3.02) [1.07]	-4.56 (2.90) [1.02]	-11.26 (5.39) [1.91]
Utw aff. neut.-aff. female	-3.33 (3.02) [1.07]	-4.70 (3.06) [1.08]	-10.80 (4.51) [1.60]
Tw aff. neut.-aff. female	-2.94 (3.13) [1.11]	-5.06 (3.33) [1.18]	-10.64 (4.98) [1.76]
Probe neut. neut.-aff. fem.	-3.11 (3.32) [1.17]	-4.93 (3.49) [1.24]	-10.77 (4.92) [1.74]
Utw neut. neut.-aff. female	-2.67 (3.26) [1.15]	-4.66 (3.44) [1.22]	-10.39 (6.24) [2.21]
Tw neut. neut.-aff. female	-2.54 (3.33) [1.18]	-4.65 (3.15) [1.11]	-11.34 (5.51) [1.95]

Note. Scores are presented in mean (standard deviation, SD) [standard error of the mean, SEM]. [1] Amplitudes are averaged over conditions (affective vs. neutral). Probe = Untrustworthy-probe. Utw = Truthful untrustworthy. Tw = Truthful trustworthy. Aff. = affective. Neut. = neutral. For statistical results and additional significant higher-order interactions revealed by post-hoc explorations, see Table S4.

Table S4. Additional statistical results for significant interaction effects on N2 amplitudes revealed by post-hoc analyses based on different quantifications.

	Mean amplitude[1]	Baseline-to-Peak amplitude[2]	Peak-to-Peak amplitude[3]
Condition × Gender × Task sequence	$F_{(1,26)} = 6.04$, $p = .02$, $\eta_p^2 = 0.19$		
Picture type × Condition × Position × Task sequence		$F_{(6,156)} = 3.75$, $p < .01$, $\eta_p^2 = 0.13$, $\varepsilon = .45$	
Picture type × Condition × Position			$F_{(6,156)} = 4.46$, $p = .01$, $\eta_p^2 = .15$, $\varepsilon = .44$
Picture type × Condition × Position × Task sequence			$F_{(6,156)} = 5.60$, $p < .01$, $\eta_p^2 = .18$, $\varepsilon = .44$

Note. [1] The remaining main effects, two-way interactions and all higher-order interactions were not significant, Fs ≤ 2.69, ps ≥ .05, $\eta_p^2 \leq .09$. [2] The remaining main effects, two-way interactions and all higher-order interactions were not significant, Fs ≤ 4.12, ps ≥ .05, $\eta_p^2 \leq .14$. [3] The remaining main effects, two-way interactions and all higher-order interactions were not significant, Fs ≤ 3.17, ps ≥ .05, $\eta_p^2 \leq .11$.

Table S5. Pearson correlation between self-reported ratings of trustworthiness and picture-wise N2 amplitudes at FCz (N = 30).

		Self-rating					
		utw-probe affective	tw affective	utw affective	utw-probe neutral	tw neutral	utw neutral
Mean N2 amplitude	Probe affective	*r* = -.33+	*r* = -.16	*r* = -.13	*r* = -.25	*r* = -.13	*r* = -.04
	Tw affective	*r* = -.30	*r* = -.11	*r* = -.11	*r* = -.20	*r* = .02	*r* = .01
	Utw affective	*r* = -.26	*r* = .03	*r* = -.11	*r* = -.08	*r* = .02	*r* = .04
	Probe neutral	*r* = -.40*	*r* = -.14	*r* = -.14	*r* = -.19	*r* = -.06	*r* = .02
	Tw neutral	*r* = -.31+	*r* = -.15	*r* = -.10	*r* = -.21	*r* = -.07	*r* = .06
	Utw neutral	*r* = -.32+	*r* = .04	*r* = -.08	*r* = -.08	*r* = .07	*r* = .05
Baseline-to-peak N2 amplitude	Probe affective	*r* = -.28	*r* = -.06	*r* = -.10	*r* = -.14	*r* = -.12	*r* = .12
	Tw affective	*r* = -.21	*r* = .02	*r* = -.13	*r* = -.04	*r* = .07	*r* = .12
	Utw affective	*r* = -.24	*r* = .09	*r* = -.07	*r* = -.05	*r* = .05	*r* = .12
	Probe neutral	*r* = -.30	*r* = -.01	*r* = -.14	*r* = -.09	*r* = -.00	*r* = .10
	Tw neutral	*r* = -.25	*r* = -.03	*r* = -.12	*r* = -.13	*r* = -.02	*r* = .12
	Utw neutral	*r* = -.23	*r* = .17	*r* = -.09	*r* = .02	*r* = .10	*r* = .11
Peak-to-Peak N2 amplitude	Probe affective	*r* = -.03	*r* = .10	*r* = .04	*r* = .11	*r* = -.18	*r* = .41*
	Tw affective	*r* = -.05	*r* = .18	*r* = .03	*r* = .16	*r* = -.14	*r* = .41*
	Utw affective	*r* = -.08	*r* = .19	*r* = .04	*r* = .06	*r* = -.09	*r* = .32+
	Probe neutral	*r* = -.08	*r* = .22	*r* = .00	*r* = .13	*r* = -.11	*r* = .37*
	Tw neutral	*r* = -.06	*r* = .15	*r* = -.03	*r* = .12	*r* = -.13	*r* = .35
	Utw neutral	*r* = -.08	*r* = .28	*r* = .00	*r* = .13	*r* = -.12	*r* = .31

Note. + The correlation is marginally significant ($p < .10$). * The correlation is significant at an alpha level of .05 (two-tailed). Probe = Untrustworthy-probe. Utw = Truthful untrustworthy. Tw = Truthful trustworthy. Trustworthiness was rated using a Likert scale from 0 = not at all trustworthy to 7 = very strongly trustworthy. The N2 amplitude is measured in microvolt (μV).

Table S6. Percentage of correct responses (%) and mean response times (ms) in correct trials for Picture type, Condition, Task sequence and Gender of the participant.

	Percentage of correct responses	Response times
Picture type[1,2,3]	*	*
Probe	96.63 (3.60) [0.66]	353.88 (146.39) [26.73]
Utw	97.03 (3.42) [0.62]	341.94 (151.84) [27.72]
Tw	98.52 (2.25) [0.41]	321.85 (135.75) [24.79]
Condition[2,3,4]		
affective	97.96 (2.72) [0.50]	329.21 (144.61) [26.40]
neutral	96.84 (4.49) [0.82]	349.23 (157.52) [28.76]
Task sequence[1,3,4]		
Affective-neutral	97.44 (2.51) [0.65]	354.07 (168.21) [43.43]
Neutral-affective	97.35 (3.05) [0.79]	324.37 (114.69) [29.61]
Gender of participant[1,2,4]		
Male	96.79 (3.42) [0.88]	326.36 (174.66) [45.10]
Female	98.00 (1.78) [0.46]	352.08 (105.16) [27.15]

Note. Scores are presented in mean (standard deviation, SD) [standard error of the mean, SEM]. * Significant at $\alpha = .05$. [1] Scores are averaged over conditions (affective vs. neutral). [2] Scores are averaged over task sequence (affective-neutral vs. neutral-affective). [3] Scores are averaged over gender of the participant. [4] Scores are averaged over picture types. Probe = Untrustworthy-probe. Utw = Truthful untrustworthy. Tw = Truthful trustworthy.

Table S7. Percentage of correct responses (%) and mean response times (ms) in correct trials for interactions of Picture type, Condition, Task sequence and Gender of the participant.

	Percentage of correct responses	Response times
Picture type × Condition[1,2]		
Probe aff.	96.80 (5.11) [0.93]	347.70 (163.15) [29.79]
Utw aff.	97.73 (3.39) [0.62]	335.34 (156.63) [28.60]
Tw aff.	99.33 (1.42) [0.26]	304.60 (126.95) [23.18]
Probe neut.	96.47 (5.63) [1.03]	360.06 (161.52) [29.49]
Utw neut.	96.33 (5.29) [0.97]	348.55 (165.47) [30.21]
Tw neut.	97.71 (4.02) [0.74]	339.10 (160.56) [29.31]
Picture type × Task sequence[2,3]	+	*
Probe aff.-neut.	96.07 (3.71) [0.96]	362.93 (177.18) [45.75]
Utw aff.-neut.	97.25 (3.31) [0.85]	371.27 (175.21) [45.24]
Tw aff.-neut.	99.01 (1.18) [0.30]	328.01 (155.62) [40.18]
Probe neut.-aff.	97.20 (3.51) [0.91]	344.83 (113.23) [29.24]
Utw neut.-aff.	96.81 (3.63) [0.94]	312.61 (123.36) [31.85]
Tw neut.-aff.	98.03 (2.93) [0.76]	315.68 (117.79) [30.41]
Picture type × Gender[1,3]		
Probe male	96.07 (4.23) [1.09]	340.04 (181.79) [46.94]
Utw male	96.02 (4.03) [1.04]	331.24 (187.22) [48.34]
Tw male	98.19 (2.62) [0.68]	307.81 (159.40) [41.16]
Probe female	97.20 (2.86) [0.74]	367.72 (104.55) [26.99]
Utw female	98.04 (2.41) [0.62]	352.65 (111.63) [28.82]
Tw female	98.75 (1.87) [0.48]	335.89 (111.11) [28.69]
Condition × Task sequence[2,4]	*	*
Aff. aff.-neut.	96.71 (3.34) [0.86]	368.54 (168.04) [43.39]
Neut. aff.-neut.	98.17 (2.42) [0.62]	339.61 (181.37) [46.83]
Aff. neut.-aff.	99.20 (0.92) [0.24]	289.89 (108.48) [28.01]
Neut. neut.-aff.	95.50 (5.66) [1.46]	358.86 (135.28) [34.93]
Condition × Gender[1,4]		
Aff. male	97.69 (3.26) [0.84]	328.17 (185.79) [47.97]
Neut. male	95.90 (5.72) [1.48]	324.55 (176.66) [45.61]
Aff. female	98.22 (2.13) [0.55]	330.25 (93.81) [24.22]
Neut. female	97.77 (2.69) [0.69]	373.91 (137.41) [35.48]
Task sequence × Gender[3,4]		
Aff.-neut. male	97.33 (2.91) [1.03]	357.18 (210.02) [74.25]
Neut.-aff. male	96.18 (4.07) [1.54]	291.14 (130.40) [49.29]
Aff.-neut. female	97.57 (2.19) [0.83]	350.52 (120.54) [45.56]
Neut.-aff. female	98.37 (1.36) [0.48]	353.46 (98.28) [34.75]

Note. Scores are presented in mean (standard deviation, SD) [standard error of the mean, SEM]. * Significant at $\alpha = .05$. + marginally significant at $\alpha = .10$. [1] Scores are averaged over task sequence (affective-neutral vs. neutral-affective). [2] Scores are averaged over gender. [3] Scores are averaged over conditions (affective vs. neutral). [4] Scores are averaged over picture types. Probe = Untrustworthy-probe. Utw = Truthful untrustworthy. Tw = Truthful trustworthy. Aff. = affective. Neut. = neutral.

Table S8A. Percentage of correct responses (%) and mean response times (ms) in correct trials for higher-order interactions of Picture type, Condition, Task sequence and Gender of the participant.

	Percentage of correct responses	Response times
Picture type × Condition × Task sequence[1]	*	
Probe aff. aff.-neut.	94.13 (6.12) [1.58]	385.68 (189.68) [48.98]
Utw aff. aff.-neut.	96.53 (4.31) [1.11]	389.73 (178.20) [46.01]
Tw aff. aff.-neut.	99.47 (1.41) [0.36]	330.21 (147.57) [38.10]
Probe neut. aff.-neut.	98.00 (3.55) [0.92]	340.18 (191.32) [49.40]
Utw neut. aff.-neut.	97.96 (2.89) [0.75]	352.81 (189.79) [49.00]
Tw neut. aff.-neut.	98.56 (2.03) [0.52]	325.82 (176.57) [45.59]
Probe aff. neut.-aff.	99.47 (1.19) [0.31]	309.72 (126.76) [32.73]
Utw aff. neut.-aff.	98.93 (1.49) [0.38]	280.95 (112.81) [29.13]
Tw aff. neut.-aff.	99.20 (1.47) [0.38]	278.99 (101.01) [26.08]
Probe neut. neut.-aff.	94.93 (6.92) [1.79]	379.94 (128.82) [33.26]
Utw neut. neut.-aff.	94.69 (6.63) [1.71]	344.28 (143.74) [37.11]
Tw neut. neut.-aff.	96.86 (5.28) [1.36]	352.37 (147.80) [38.16]
Picture type × Condition × Gender[2]		
Probe aff. male	96.93 (5.55) [1.43]	345.75 (208.46) [53.82]
Utw aff. male	96.93 (4.33) [1.12]	332.36 (191.99) [49.57]
Tw aff. male	99.20 (1.47) [0.38]	305.39 (162.93) [42.07]
Probe neut. male	95.20 (7.28) [1.88]	333.33 (182.54) [47.13]
Utw neut. male	95.10 (6.40) [1.65]	330.11 (191.13) [49.35]
Tw neut. male	97.39 (4.36) [1.13]	310.23 (167.13) [43.15]
Probe aff. female	96.67 (4.82) [1.25]	348.64 (108.09) [27.91]
Utw aff. female	98.53 (1.92) [0.50]	338.31 (118.08) [30.49]
Tw aff. female	99.47 (1.41) [0.36]	303.81 (82.69) [21.35]
Probe neut. female	97.73 (3.01) [0.78]	386.80 (138.53) [35.77]
Utw neut. female	97.55 (3.72) [0.96]	366.98 (139.50) [36.02]
Tw neut. female	98.04 (3.78) [0.98]	367.96 (153.89) [39.73]
Condition × Task sequence × Gender[3]		
Aff. aff.-neut. male	96.42 (4.05) [1.43]	385.72 (216.45) [76.53]
Neut. aff.-neut. male	98.24 (2.23) [0.81]	328.65 (213.89) [75.62]
Aff. neut.-aff. male	99.14 (1.00) [0.38]	262.40 (128.15) [48.44]
Neut. neut.-aff. male	93.21 (7.37) [2.79]	319.87 (139.28) [52.64]
Aff. aff.-neut. female	97.05 (2.58) [0.97]	348.90 (101.92) [38.52]
Neut. aff.-neut. female	98.09 (2.74) [1.04]	352.13 (151.80) [57.37]
Aff. neut.-aff. female	99.25 (0.90) [0.32]	313.94 (89.69) [31.71]
Neut. neut.-aff. female	97.49 (2.79) [0.99]	392.98 (130.86) [46.27]

Note. Scores are presented in mean (standard deviation, SD) [standard error of the mean, SEM]. * Significant at $\alpha = .05$. [1] Scores are averaged over gender. [2] Scores are averaged over task sequence (affective-neutral vs. neutral-affective). [3] Scores are averaged over picture types. Probe = Untrustworthy-probe. Utw = Truthful untrustworthy. Tw = Truthful trustworthy. Aff. = affective. Neut. = neutral. For statistical results and additional significant higher-order interactions revealed by post-hoc explorations for behavioral data, see Table S12.

Table S8B. Percentage of correct responses (%) and mean response times (ms) in correct trials for higher-order interactions of Picture type, Condition, Task sequence and Gender of the participant.

	Percentage of correct responses	Response times
Picture type × Task sequence × Gender[1]		
Probe. aff.-neut. male	96.12 (4.05) [1.43]	365.30 (222.16) [78.55]
Utw. aff.-neut. male	96.60 (3.95) [1.40]	373.75 (217.77) [76.99]
Tw aff.-neut. male	99.26 (1.15) [0.41]	332.50 (192.31) [67.99]
Probe neut.-aff. male	96.00 (4.76) [1.80]	311.17 (133.07) [50.30]
Utw. neut.-aff. male	95.35 (4.32) [1.63]	282.65 (145.94) [55.16]
Tw neut.-aff. male	97.19 (3.43) [1.30]	279.59 (119.99) [45.35]
Probe aff.-neut. female	96.00 (3.61) [1.36]	360.22 (125.10) [47.28]
Utw. aff.-neut. female	97.98 (2.48) [0.94]	368.44 (127.61) [48.23]
Tw. aff.-neut. female	98.73 (1.23) [0.47]	322.89 (115.36) [43.60]
Probe neut.-aff. female	98.25 (1.58) [0.56]	374.29 (91.33) [32.29]
Utw neut.-aff. female	98.10 (2.52) [0.89]	338.83 (102.45) [36.22]
Tw neut.-aff. female	98.77 (2.39) [0.85]	347.26 (113.87) [40.26]
Picture type × Condition × Task sequence × Gender		
Probe aff. aff.-neut. male	94.50 (6.82) [2.41]	407.24 (249.20) [88.11]
Utw aff. aff.-neut. male	95.25 (5.34) [1.89]	400.62 (211.75) [74.87]
Tw aff. aff.-neut. male	99.50 (1.41) [0.50]	349.30 (194.51) [68.77]
Probe neut. aff.-neut. male	97.75 (4.06) [1.44]	323.37 (223.66) [79.07]
Utw neut. aff.-neut. male	97.96 (2.89) [1.02]	346.88 (226.19) [79.97]
Tw neut. aff.-neut. male	99.02 (1.48) [0.52]	315.70 (199.93) [70.68]
Probe aff. neut.-aff. male	99.71 (0.76) [0.29]	277.63 (135.99) [51.40]
Utw aff. neut.-aff. male	98.86 (1.57) [0.60]	254.35 (142.76) [53.96]
Tw aff. neut.-aff. male	98.83 (1.56) [0.60]	255.22 (110.88) [41.91]
Probe neut. neut.-aff. male	92.29 (9.27) [3.50]	344.70 (138.23) [52.25]
Utw neut. neut.-aff. male	91.84 (7.90) [2.99]	310.95 (157.31) [59.46]
Tw neut. neut.-aff. male	95.52 (5.85) [2.21]	303.97 (135.84) [51.34]
Probe aff. aff.-neut. female	93.71 (5.71) [2.16]	361.04 (100.87) [38.13]
Utw aff. aff.-neut. female	98.00 (2.31) [0.87]	377.28 (146.45) [55.35]
Tw aff. aff.-neut. female	99.43 (1.51) [0.57]	308.39 (75.03) [28.36]
Probe neut. aff.-neut. female	98.29 (3.15) [1.19]	359.39 (161.99) [61.23]
Utw neut. aff.-neut. female	97.96 (3.12) [1.18]	359.60 (155.75) [58.87]
Tw neut. aff.-neut. female	98.04 (2.53) [0.96]	337.39 (160.69) [60.73]
Probe aff. neut.-aff. female	99.25 (1.49) [0.53]	337.80 (119.82) [42.36]
Utw aff. neut.-aff. female	99.00 (1.51) [0.54]	304.21 (81.58) [28.84]
Tw aff. neut.-aff. female	99.50 (1.41) [0.50]	299.80 (93.86) [33.18]
Probe neut. neut.-aff. female	97.25 (3.01) [1.07]	410.78 (120.34) [42.55]
Utw neut. neut.-aff. female	97.19 (4.36) [1.54]	373.44 (134.26) [47.67]
Tw neut. neut.-aff. female	98.04 (4.80) [1.70]	394.72 (153.23) [54.18]

Note. Scores are presented in mean (standard deviation, SD) [standard error of the mean, SEM]. * Significant at $\alpha = .05$. [1] Scores are averaged over conditions (affective vs. neutral). Probe = Untrustworthy-probe. Utw = Truthful untrustworthy. Tw = Truthful trustworthy. Aff. = affective. Neut. = neutral. For statistical results and additional significant higher-order interactions revealed by post-hoc explorations for behavioral data, see Table S9.

Table S9. Additional statistical results for significant interaction effects on behavioral data revealed by post-hoc analyses based on different quantifications.

	Percentage of correct responses[1]	Response times[2]
Condition × Task sequence	$F_{(1,26)} = 11.86$, $p < .01$, $\eta_p^2 = 0.31$	$F_{(1,26)} = 8.09$, $p = .01$, $\eta_p^2 = 0.24$
Picture type × Condition × Task sequence	$F_{(2,52)} = 6.01$, $p < .01$, $\eta_p^2 = 0.19$, $\varepsilon = .87$	

Note. [1] The remaining main effects, two-way interactions and all higher-order interactions were not significant, $F\text{s} \leq 2.61$, $p\text{s} \geq .12$, $\eta_p^2 \leq .09$. [2] The remaining main effects, two-way interactions and all higher-order interactions were not significant, $F\text{s} \leq 1.53$, $p\text{s} \geq .23$, $\eta_p^2 \leq .06$.
